# Phytochemical Profiles and Antioxidant Activity of *Elaeagnus multiflora* Thunb. Leaf and Fruit Extracts in Chemical and Erythrocyte-Derived Membrane Models

**DOI:** 10.3390/antiox15101279

**Published:** 2026-10-02

**Authors:** Katarzyna Męczarska, Wiktoria Tarasiuk, Dariusz Jedrejek, Anna Bieniek, Sabina Lachowicz-Wiśniewska, Dorota Bonarska-Kujawa, Sylwia Cyboran-Mikołajczyk

**Affiliations:** 1 Department of Physics and Biophysics, Wrocław University of Environmental and Life Sciences, Norwida Str. 25, 50-375 Wrocław, Poland; wiktoria.tarasiuk@upwr.edu.pl (W.T.); dorota.bonarska-kujawa@upwr.edu.pl (D.B.-K.); sylwia.cyboran@upwr.edu.pl (S.C.-M.); 2 Department of Phytochemistry, Institute of Soil Science and Plant Cultivation—State Research Institute, Czartoryskich 8 Street, 24-100 Puławy, Poland; djedrejek@iung.pulawy.pl; 3 Department of Agroecosystems and Horticulture, University of Warmia and Mazury in Olsztyn, Łódzki 3 Sq, 10-727 Olsztyn, Poland; anna.bieniek@uwm.edu.pl; 4 Department of Medicine and Health Sciences, University of Kalisz, 2 W. Boguslawskiego Square, 62-800 Kalisz, Poland; 5 Department of Biotechnology and Food Analysis, Wroclaw University of Economics and Business, 53-345 Wroclaw, Poland

**Keywords:** polyphenols, oxidative stress, erythrocyte membrane, membrane lipid peroxidation, hydrolysable tannins, kaempferol glycosides, fluorescence spectroscopy, membrane interactions, biological antioxidants, UHPLC–HR–QTOF–MS/MS

## Abstract

*Elaeagnus multiflora* Thunb. is a rich source of structurally diverse phytochemicals, yet the relationship between organ-specific composition, antioxidant activity, and protection of biological membranes remains insufficiently understood. In this study, XAD-16-purified phenolic-rich fractions obtained from leaves and fruits of the ‘Marianna’ cultivar were characterized using UHPLC–CAD–HR–QTOF–MS/MS and UHPLC–UV, and their antioxidant properties were evaluated using DPPH, CUPRAC, and ORAC assays as well as erythrocyte membranes and a lipid model of these membranes. Hereafter, these fractions are referred to as leaf and fruit extracts. The leaf extract was dominated by flavonol glycosides, with kaempferol derivatives as the major constituents, whereas the fruit extract contained higher contributions from gallic acid derivatives, flavan-3-ols, and hydrolysable tannins. The fruit extract showed stronger DPPH and CUPRAC activities than the leaf extract (IC_50_: 12.7 ± 0.7 vs. 63.5 ± 5.4 and 11.4 ± 2.0 vs. 33.1 ± 1.2 µg/mL, respectively), whereas the leaf extract exhibited greater ORAC activity (2.2 ± 0.3 vs. 6.0 ± 0.7 µg/mL). In AAPH-treated liposomes prepared from erythrocyte membrane lipids, the fruit extract showed stronger protection against lipid oxidation (IC_50_ = 3.8 ± 1.2 vs. 9.5 ± 1.1 µg/mL). Neither extract increased erythrocyte hemolysis relative to the control. Fluorescence measurements indicated more pronounced effects of the fruit extract on membrane-associated parameters, while the leaf extract produced no significant changes in DPH anisotropy or Laurdan generalized polarization. The results demonstrate that antioxidant efficacy depends on phytochemical composition and experimental context. Although the stronger DPPH and CUPRAC responses of the fruit fraction co-varied with its greater protection in AAPH-treated RBCL liposomes, this relationship was not reproduced across the remaining membrane models, and the superior ORAC response of the leaf fraction did not translate into greater membrane protection.

## 1. Introduction

The *Elaeagnaceae* family comprises woody plants distributed predominantly across temperate and subtropical regions of the Northern Hemisphere. Its major genera include *Elaeagnus, Hippophae*, and *Shepherdia*, with *Elaeagnus* representing the largest genus and comprising approximately 60 species [1]. Species of this genus are characterized by diverse phytochemical profiles containing phenolic acids, flavonoids, proanthocyanidins, carotenoids, and other specialized metabolites associated with antioxidant activity [2,3,4]. Importantly, the distribution of these compounds varies considerably among plant organs as a consequence of organ-specific secondary metabolism. Fruits and leaves may therefore represent chemically and functionally distinct matrices, with leaves constituting a particularly rich source of flavonoid glycosides and other phenolic compounds [2,3,4,5].

Among *Elaeagnus* species, *Elaeagnus multiflora* Thunb., commonly known as goumi or cherry silverberry, has attracted increasing interest because of the phytochemical richness and antioxidant properties of its fruits and leaves. The fruits contain procyanidins and flavonoids together with lipophilic antioxidants such as lycopene and α-tocopherol and hydrophilic constituents including vitamin C and organic acids [5]. Previous studies have demonstrated antioxidant activity of *E. multiflora* extracts in chemical radical scavenging assays and have also reported anti-inflammatory effects in cellular models [6]. Detailed phytochemical investigations have further demonstrated marked differences between fruits and leaves, including the predominance of flavonol derivatives in leaves and organ-dependent distributions of other polyphenolic and isoprenoid constituents [2,3,4,5]. Nevertheless, the extent to which these compositional differences determine antioxidant performance in biological membrane systems remains insufficiently characterized.

Plant-derived phenolic compounds can exert antioxidant effects through several mechanisms, including radical scavenging, electron or hydrogen donation, and interactions with biological interfaces [7]. High-resolution chromatographic and mass-spectrometric approaches allow complex phytochemical mixtures to be characterized in considerable detail [8]; however, chemical composition alone does not necessarily predict antioxidant efficacy under biologically relevant conditions. Likewise, individual antioxidant assays probe different reaction mechanisms and physicochemical environments. Consequently, the combined use of complementary chemical assays and membrane-based experimental models can provide a more informative assessment of antioxidant behavior than either approach alone.

Erythrocytes and erythrocyte-derived membrane systems are particularly useful models for studying oxidative membrane damage [9]. Red blood cells are continuously exposed to high oxygen concentrations and contain abundant polyunsaturated membrane lipids and redox-active hemoglobin, while lacking nuclei and mitochondria. Their antioxidant defense system includes reduced glutathione (GSH), which participates in the removal of reactive species and in the maintenance of cellular redox balance. Depletion of GSH is therefore an important indicator of oxidative stress and may accompany damage to erythrocyte membrane components [9,10]. In addition to intact erythrocyte-derived membranes, liposomes prepared from erythrocyte membrane lipids provide a complementary protein-free system in which antioxidant effects on the lipid fraction can be examined separately from those occurring in the structurally more complex lipid–protein environment of erythrocyte membranes. Protective effects of plant-derived polyphenolic extracts in erythrocyte and membrane models have previously been demonstrated for several phytochemical matrices [9,11].

Complementary oxidative stress models can further distinguish antioxidant effects under different mechanisms of membrane damage. Thermal decomposition of 2,2′-azobis(2-methylpropionamidine) dihydrochloride (AAPH) generates peroxyl radicals capable of initiating lipid peroxidation and damaging membrane proteins, ultimately affecting erythrocyte membrane integrity and function [12,13]. Ultraviolet radiation provides a mechanistically distinct physical stressor capable of inducing photochemical damage to membrane lipids and proteins [14]. The use of both AAPH- and UVC-induced oxidation, together with protein-containing erythrocyte membranes and lipid-only membrane models, therefore, enables antioxidant protection to be examined across complementary oxidative and structural environments.

The present study aimed to compare the phytochemical profiles and antioxidant properties of leaf and fruit extracts from the ‘Marianna’ cultivar of *E. multiflora* Thunb. We hypothesized that the marked organ-specific differences in phytochemical composition would result in distinct, assay-dependent antioxidant responses and that antioxidant activity measured in chemical systems would not necessarily predict protection in biological membrane models. Accordingly, the extracts were characterized using UHPLC–CAD–HR–QTOF–MS/MS and UHPLC–UV and evaluated using complementary DPPH, ORAC, and CUPRAC assays. Their protective effects were subsequently investigated in erythrocyte membranes and liposomes prepared from erythrocyte membrane lipids exposed to AAPH- and UVC-induced oxidative stress. Changes in intracellular GSH and erythrocyte hemolysis were assessed in parallel, while DPH and Laurdan fluorescence measurements were used to probe extract-associated changes in the hydrophobic and interfacial regions of the membrane. This integrated approach was designed to determine how organ-specific phytochemical composition relates to antioxidant activity across chemical and membrane-based experimental systems.

## 2. Materials and Methods

### 2.1. Chemicals and Reagents

Folin–Ciocalteu reagent, 5-caffeoylquinic acid (≥95%), catechin (≥98%), gallic acid (≥98%), rutin (≥95%), and acetonitrile and formic acid (both MS grade) were obtained from Merck (Darmstadt, Germany). Ultrapure water was prepared with a Milli-Q purification system (Millipore, Burlington, MA, USA). The fluorescent probes 6-dodecanoyl-2-dimethylaminonaphthalene (Laurdan), CAS No.: 74515-25-6; 1,6-diphenyl-1,3,5-hexatriene (DPH), CAS No.: 484049-04-9; 1-(4-trimethylammoniumphenyl)-6-phenyl-1,3,5-hexatrienep-toluenesulfonate (TMA-DPH), CAS No.: 115534-33-3; 3′,6′-dihydroxyspirobenzofuran (Fluorescein sodium salt) CAS No.: 2321-07-5; 2,2-diphenyl-1-picrylhydrazyl (DPPH), CAS No.: 1898-66-4; the 2,2′-azobis(2-methylpropionamidine) dihydrochloride (AAPH), CAS No.: 2997-92-4; (±)-6-hydroxy-2,5,7,8-tetramethylchroman-2-carboxylic acid (Trolox^®^) CAS No.: 53188-07-1; copper(II) chloride (CuCl_2_) CAS No.: 7447-39-4; 2,9-dimethyl-1,10-phenanthroline (neocuproine) CAS No.: 484-11-7. Heparin was obtained from Polfa Warszawa (Warszawa, Poland). Substances used for the preparation of buffers included NaCl (Avantor Performance Materials, Gliwice, Poland), Na_2_HPO_4_·H_2_O, NaH_2_PO_4_·H_2_O, EDTA, and Tris (Chempur, Piekary Śląskie, Poland). Ethanol was purchased from Avantor Performance Materials, Gliwice, Poland. Thiobarbituric acid (TBA) and immersion oil were obtained from Honeywell Fluka (Charlotte, NC, USA), and trichloroacetic acid (TCA) was purchased from Eurochem BGD (Tarnów, Poland).

### 2.2. Plant Material and Preparation of Extracts

Fruits and leaves of *Elaeagnus multiflora* Thunb. cv. ‘Marianna’ were collected in June 2023, at the stage of optimal fruit ripeness, from the long-term experimental plant collection of the Department of Agroecosystems and Horticulture, University of Warmia and Mazury in Olsztyn, Poland (53°50′ N, 20°31′ E). The cultivar corresponds to the former UWM breeding accession 9-84-1996 (SiA), which originated from seeds of the 01-1999 (si0) biotype obtained in 2005 [2,4]. The plants were mature adult shrubs grown under field conditions in the experimental collection. They were established at a spacing of 4 × 2 m in Albic Luvisol (Arenic) soil developed from clay, with a pH of approximately 6.8 in KCl. The shrubs began fruiting in the third year after establishment. The cultivar ‘Marianna’ is registered in the Polish National Register of Varieties maintained by the Research Center for Cultivar Testing (COBORU) under registration No. S 288. The botanical identity of the plant material was confirmed by Prof. Anna Bieniek, Department of Agroecosystems and Horticulture, University of Warmia and Mazury in Olsztyn, Poland, who also provided the material used in this study. A total of 5 kg of plant material was collected, and fruits and leaves were processed separately. Fresh fruits were mechanically crushed prior to extraction. Leaves were freeze-dried using an ALPHA 1–2 LD plus freeze-dryer (Martin Christ Gefriertrocknungsanlagen GmbH, Osterode am Harz, Germany) and subsequently milled. Both plant materials were extracted with 70% (*v*/*v*) ethanol at a plant material-to-solvent ratio of 1:3 (*w*/*v*) using ultrasound-assisted extraction for 30 min at 30 °C in a Sonic 10 ultrasonic bath (Polsonic, Warsaw, Poland), operated at a frequency of 40 kHz and a nominal ultrasonic power of 2 × 400 W (effective power 200 W). The extraction procedure was performed three times for each plant material. After each extraction cycle, the suspensions were centrifuged at 10,000× *g* for 10 min, and the resulting supernatants were collected. The ethanolic extracts were subsequently concentrated under reduced pressure using an R-215 Rotavapor system (Büchi, Flawil, Switzerland) to remove ethanol. Prior to purification, the concentrated extracts were acidified with hydrochloric acid to pH 2.0 and centrifuged to remove insoluble material. The extracts were then purified using Amberlite XAD-16 polymeric adsorbent resin (Brenntag, Poland). Before the sample application, the resin was conditioned with methanol and equilibrated with water. After adsorption of the extracts, the resin was washed with water to remove sugars, organic acids, and other highly polar constituents. Phenolic compounds retained on the resin were subsequently desorbed with 80% (*v*/*v*) ethanol. The phenolic-rich eluates were collected and concentrated under reduced pressure to remove ethanol. The purified extracts were then freeze-dried using an ALPHA 1–2 LD plus freeze-dryer and stored under refrigerated conditions until further analysis. For chromatographic analyses, the freeze-dried extracts were dissolved in 20% (*v*/*v*) methanol containing 0.1% formic acid to a final concentration of 5 mg/mL. The stock solutions were stored at −20 °C and, immediately before UHPLC analysis, were appropriately diluted and centrifuged at 12,000× *g* for 10 min at 8 °C. For antioxidant and membrane-based biological assays, separate stock solutions of the freeze-dried extracts were prepared in dimethyl sulfoxide (DMSO) at 10 mM and diluted with the appropriate assay medium or buffer to obtain the required working concentrations. Corresponding vehicle controls contained the same amount of DMSO as the extract-treated samples. Two independently prepared extracts were obtained for each plant organ. The resulting XAD-16-purified, phenolic-rich fractions are hereafter referred to as the leaf and fruit extracts.

### 2.3. Red Blood Cells and Model Membranes

The biological material consisted of human erythrocytes isolated from residual blood material remaining after the routine preparation of red blood cell concentrates supplied by the Regional Center for Blood Donation and Blood Treatment, named after Tadeusz Dorobisz in Wrocław, under a scientific cooperation agreement with the Wrocław University of Environmental and Life Sciences. The investigators did not recruit blood donors, did not perform blood collection for the purposes of the present study, had no direct contact with donors, and received no direct identifiers, donor identification numbers, donor codes, coding keys, or other information that would enable linkage of the samples to individual donors. The only information accompanying the samples was the blood group and the date of blood collection. Thus, the investigators had no means to identify or re-identify individual donors. According to the Polish Act of 5 December 1996 on the Professions of Physician and Dentist (consolidated text: Journal of Laws of 2026, item 37), Article 21(4) defines research on biological material collected from a person for scientific purposes as a medical experiment, while Article 29(1) requires prior approval by a bioethics committee for a medical experiment. In the present study, blood was collected by the blood donation center as part of routine blood donation, independently of the present research, and only residual material was subsequently provided to the investigators under the institutional cooperation agreement; no biological material was collected from donors specifically for this study. Consequently, the study did not constitute a medical experiment as defined by Article 21(4), and prior approval by a bioethics committee was not required under the applicable Polish legislation.

Cell isolation was performed according to a previously described procedure. Erythrocyte membranes (RBC ghosts) were prepared from fresh blood using the method originally described by Dodge et al. [15]. Protein concentration in the preparation was determined by the Lowry method [16] and was approximately 100 µg/mL. Lipids were extracted from erythrocytes according to the procedure established by Maddy [17], yielding erythrocyte-derived lipid extracts to a final concentration of 10 mg/mL. For the preparation of large unilamellar vesicles (LUVs), lipids were evaporated under a stream of nitrogen and subsequently dried under reduced pressure using a rotary evaporator. The resulting lipid film was hydrated in a buffer appropriate for the given analytical procedure and homogenized by vortexing for several minutes to yield multilamellar vesicles (MLVs). The MLV suspension was then subjected to extrusion through polycarbonate membranes with pore diameters of 200 nm followed by 100 nm (Whatman^®^ Nuclepore™, GE Healthcare, Chicago, IL, USA), using an automated pressure extruder (Lipex, Evonik Industries AG, Essen, Germany).

### 2.4. Phytochemical Analyses

#### 2.4.1. Qualitative Liquid Chromatography High-Resolution Mass-Spectrometric Analysis

High-resolution MS/MS data were obtained using a Bruker Impact II HD Q-TOF mass spectrometer (Bruker, Billerica, MA, USA) coupled to an Ultimate 3000 RS UHPLC system (Thermo Scientific, Waltham, MA, USA). The MS was operated in electrospray ionization and both polarity modes, with the following settings: mass scan range 80–1500 *m*/*z*; capillary voltage 4.5 kV (ESI+) or 3.0 kV (ESI−); nebulizer and dry gas (N_2_) 0.7 bar and 6 L/min, respectively; dry gas temperature 200 °C. The MS/MS spectra were acquired using variable collision energy in the range from 7.0 to 70 eV. The acquired data were calibrated internally with sodium formate (10 mM solution in 50% 2-propanol), which was injected into the ion source at the beginning of each analysis.

The UHPLC system was additionally equipped with a diode array detector (DAD) and a corona-charged aerosol detector (CAD). The UV absorbance was measured in the range of 200–600 nm (5 nm bandwidth). The acquisition frequency of both the DADs and CADs was set to 10 Hz. Chromatographic separation was performed on a HSS C18 column (100 × 2.1 mm, 1.7 µm, Waters, Milford, MA, USA) equipped with a precolumn. A 27 min linear gradient (02 → 40%) of acetonitrile–water (both acidified with 0.1% formic acid) was applied at a flow rate of 0.4 mL/min. The column was maintained at 45 °C. The injection volume was 2 µL.

Data acquisition and initial processing were performed using DataAnalysis 4.4 software (Bruker). Compound annotation was additionally supported using SIRIUS 6.0 [18]. Molecular formula candidates were evaluated based on accurate mass, isotope patterns, and MS/MS fragmentation trees, while tentative structural candidates were assessed using CSI:FingerID searches against the structure databases selected in the SIRIUS platform, including ChEBI, COCONUT, GNPS, KNApSAcK, LOTUS, PubChem, and SuperNatural [19]. No dedicated MS/MS spectral library matching was performed. Final annotations were assigned by integrating SIRIUS/CSI:FingerID-supported candidates with experimental MS/MS fragmentation patterns, UV–Vis spectral characteristics, chromatographic behavior, literature data, and, where available, comparison with authentic standards.

#### 2.4.2. Quantitative UHPLC-UV Analysis of Phenolics

Phenolic compounds, including flavan-3-ols, flavonols, gallic acid derivatives, and hydroxycinnamic acid derivatives, were determined using a Premier UPLC system equipped with a photodiode array detector (_λ_PDA) and a Xevo TQ-XS mass spectrometer (Waters Corporation, Milford, MA, USA). The same chromatographic column and conditions described in Section 2.4.1 were used.

Quantitative determinations were based on an external standard method, using an appropriate group standard and detection at a specific wavelength: 345 nm for flavonols (rutin), 325 nm for hydroxycinnamic acids (5-*O*-caffeoylquinic acid), 280 nm for flavanols (catechin), and 275 nm for gallic acid derivatives (gallic acid). The calibration curve range for the analytical standard was adjusted to match the specific levels of the compounds in the samples, including rutin (0.5–200 µg/mL), 5-chlorogenic acid (0.25–50 µg/mL), catechin (1–100 µg/mL), and gallic acid (0.5–75 µg/mL). Stock solutions of these substances were prepared in 20% MeOH containing 0.1% FA, then diluted as needed to prepare working solutions. All solutions were stored at –20 °C. Three to five injections were performed for each sample or standard working solution. All four curves exhibited excellent validation parameters, including R^2^ ≥ 0.999, goodness-of-fit ≤ 1%, and a limit of detection of ≤0.15 µg/mL (Table S1).

Quantitative results are expressed as milligrams of standard equivalents (eq) per gram of dry extract (d.m.).

#### 2.4.3. Total Phenolic and Condensed Tannin Content

Colorimetric measurements were performed using an Evolution 260 Bio spectrophotometer (Thermo Scientific).

Total phenolic content (TPC) was determined by the Folin–Ciocalteu method as described in the paper by Singleton et al. [20]. Absorbance was measured at 765 nm after 30 min of incubating samples at 40 °C. The TPC was determined from the linear curve for gallic acid (1–10 µg/mL; R^2^ > 0.998) and expressed as milligrams of gallic acid equivalents per gram of extract d.m.

Condensed tannin content (CTC) was determined by the vanillin method [2]. Absorbance was measured at 500 nm after 20 min in the dark. The CTC was read from the linear curve for catechin (100–1000 µg/mL; R^2^ > 0.994) and expressed as milligrams of catechin equivalents per gram of extract d.m.

### 2.5. Antioxidant Activity Assays

#### 2.5.1. DPPH Radical Scavenging Assay

Antiradical activity was measured using the DPPH method with an Agilent BioTek Epoch microplate spectrophotometer (Agilent Technologies, Santa Clara, CA, USA) according to the procedure described by Kaźmierczak et al. [21]. DPPH radical scavenging activity was assessed in 96-well plates at a final volume of 200 µL. The DPPH solution was prepared daily in ethanol to achieve an initial absorbance of approximately 1.0 at 517 nm. The DPPH solution served as the reaction control, and ethanol served as a blank. After adding the test extracts at the appropriate concentrations, the plate was incubated in the dark with shaking for 30 min, after which the absorbance was measured at 517 nm. The results were expressed as IC_50_ values (µg/mL). For all assays expressed as IC_50_, lower IC_50_ values indicate stronger antioxidant or membrane-protective activity.

#### 2.5.2. Oxygen Radical Absorbance Capacity (ORAC) Assay

Oxygen Radical Absorbance Capacity (ORAC) was determined according to the method of Ou et al. [22], with modifications. The fluorescein (FL) working solution (1 µM) was prepared by diluting 25 µL of a 1 mM fluorescein stock solution in 25 mL of phosphate buffer (75 mM, pH 7.4). AAPH (2,2′-azobis(2-methylpropionamidine) dihydrochloride) solution (1 M) was freshly prepared in the same phosphate buffer immediately before each analysis. Measurements were performed at 37 °C using a Cary Eclipse spectrofluorometer (Varian, Palo Alto, CA, USA) equipped with a 1 mL quartz cuvette. The excitation and emission wavelengths were set at 485 and 528 nm, respectively.

Three experimental conditions were distinguished for data normalization: a non-oxidized blank containing fluorescein without AAPH, an AAPH-treated control containing fluorescein and AAPH without antioxidant, and samples containing fluorescein, AAPH, and the tested extract or reference antioxidant at the appropriate concentration. In the oxidized control and antioxidant-treated samples, the oxidation reaction was initiated by the addition of 40 µL of freshly prepared 1 M AAPH solution, corresponding to a final AAPH concentration of approximately 40 mM. Fluorescence decay was recorded at 30 s intervals for 31 min.

The area under the fluorescence decay curve (AUC) was calculated from fluorescence values normalized to the initial fluorescence according to:

(1) $$AUC=\sum_{i=1}^{n-1}\frac{1}{2}(\frac{fi}{fo}+\frac{fi+1}{f0})([ti+1]-ti)$$

where $f_{0}$ is the fluorescence intensity recorded at the beginning of the measurement and $f_{i}$ is the fluorescence intensity recorded at each subsequent time point. For each antioxidant-treated sample, the net area under the curve (NetAUC) was calculated relative to the AAPH-treated control:

(2) $${AUC}_{net}={AUC}_{sample}-{AUC}_{control}$$

Antioxidant protection was subsequently expressed relative to the maximal protection represented by the non-oxidized blank:

$$Protection\;\left(\%\right)={AUC}_{sample}-\frac{{AUC}_{control}}{{AUC}_{blank}}-{AUC}_{control}\times \;100$$

where $AUC_{sample}$ represents the area under the fluorescence decay curve obtained in the presence of the tested extract or reference antioxidant, $AUC_{control}$ represents the AUC of the AAPH-treated control without antioxidant, and $AUC_{blank}$ represents the AUC of the non-oxidized fluorescein blank. A concentration–response relationship was constructed from the resulting protection values. The ORAC IC_50_ was defined as the concentration of the tested extract or reference antioxidant (µg/mL) producing 50% protection against AAPH-induced fluorescein oxidation under the applied assay conditions and was determined by linear regression of the concentration–response relationship. Accordingly, lower ORAC IC_50_ values indicate greater peroxyl radical scavenging capacity.

#### 2.5.3. Cupric Ion Reducing Antioxidant Capacity (CUPRAC) Assay

Cupric Ion Reducing Antioxidant Capacity was determined according to Apak et al. [23], with modifications adapted for a microplate format. Measurements were performed in a 96-well microplate using an Agilent BioTek Epoch microplate spectrophotometer (Agilent Technologies, Santa Clara, CA, USA). First, 180 µL of a reagent mixture containing CuCl_2_ (10 mM), neocuproine (7.0 mM), and ammonium acetate buffer (1 M, pH 7.0) in a 1:1:1 (*v*/*v*/*v*) ratio was added to each well. Then, 20 µL of the test extracts at the appropriate concentration were added (final volume: 200 µL per well). After incubating for 30 min at room temperature in the dark, the absorbance was measured at 450 nm. Results were expressed as IC_50_ values (µg/mL).

### 2.6. Biological Antioxidant Protection Assays

The antioxidant activity of the tested extracts toward erythrocyte membranes was assessed using fluorometric and spectrophotometric methods according to the method described by Cyboran et al. [24]. Ultraviolet C (UVC) radiation and 2,2′-azobis(2-methylpropionamidine) dihydrochloride (AAPH) were used as oxidative stress inducers. In the fluorometric method, the TMA-DPH probe was used, and the degree of lipid peroxidation was determined based on changes in fluorescence intensity measured at λ_ex_ = 362 nm and λ_em_ = 429 nm using a Cary Eclipse spectrofluorometer (Varian, Palo Alto, CA, USA). In the spectrophotometric method, the level of lipid peroxidation was assessed based on the concentration of malondialdehyde (MDA) determined by reaction with thiobarbituric acid (TBA) at a wavelength of 535 nm using a SPECORD^®^ 40 UV-Vis spectrophotometer (Analytik Jena AG, Jena, Germany). Antioxidant activity was expressed as the IC_50_ value, defined as the concentration of the tested sample required to inhibit lipid peroxidation by 50%. The results were compared with Trolox^®^ as the reference antioxidant standard.

### 2.7. Effects of Extracts on Erythrocytes: Hemolysis and Glutathione Content

The hemolytic activity of the extracts was determined using the method described previously by Cyboran-Mikołajczyk [25]. The measurement was performed spectrophotometrically at a wavelength of 540 nm after 1 h of incubation, in the concentration range 0–200 µg/mL, using a SPECORD^®^ 40 spectrophotometer (Analytik Jena AG, Jena, Germany).

The concentration of reduced glutathione (GSH) was determined spectrophotometrically by measuring the reaction of GSH’s sulphydryl groups with Ellman’s reagent (DTNB), in accordance with the procedure previously described by the authors [11] and the Ellman method [26], with minor modifications. A suspension of erythrocytes with a hematocrit of 10% in 0.5 M phosphate-buffered solution (pH 8.2) was incubated with the test extracts at concentrations ranging from 0 to 75 µg/mL for 1 h at 37 °C. To assess the extracts’ protection against oxidative stress, AAPH was added, and the samples were then incubated for 1 h at 37 °C. After incubation, the samples were treated with DTNB and incubated for 30 min in the dark at room temperature. The absorbance of the samples relative to the reference sample was measured at a wavelength of 412 nm using a SPECORD^®^ 40 spectrophotometer (Analytik Jena AG, Jena, Germany). The GSH concentration was determined based on the absorbance values obtained.

### 2.8. The Impact of Extract Components on the Hydrophilic and Hydrophobic Regions of Erythrocyte Membranes

The fluidity and organization of erythrocyte membranes and model lipid membranes were investigated using a fluorometric method according to the procedure previously described by Cyboran et al. [24]. Fluorescence measurements were carried out using a Cary Eclipse spectrofluorometer (Varian, Palo Alto, CA, USA) and performed at 37 °C. The experiments were performed on erythrocyte membranes suspended in phosphate buffer at pH 7.4 and on model lipid membranes prepared from lipids isolated from erythrocyte membranes. The extracts were examined at final concentrations of 0–30 µg/mL in the incubation medium. Membrane fluidity was assessed using the fluorescent probe DPH, which localizes in the hydrophobic region of the lipid bilayer. The excitation and emission wavelengths were set at λ_ex_ = 360 nm and λ_em_ = 425 nm. The fluidity of the membranes was quantified based on the fluorescence anisotropy (A) of the DPH probe calculated according to the methods described by Lakowicz [27] and Parasassi et al. [28]. Changes in membrane organization in the lipid–water interfacial region were additionally assessed using the Laurdan probe. This probe is sensitive to changes in polarity, hydration, and lipid packing near the phospholipid head-group region. Measurements were performed at an excitation wavelength of λ_ex_ = 360 nm, while fluorescence intensity was recorded at two emission wavelengths: 440 nm and 490 nm. Based on these values, Laurdan generalized polarization (GP) was calculated. An increase in GP indicates greater ordering and/or reduced hydration of the membrane lipid head region, whereas a decrease in GP reflects increased hydration, or loosening of lipid packing in this region, resulting in decreased ordering in this region.

### 2.9. Statistical Analysis

Data are presented as mean ± standard deviation (SD). Independent experimental or extraction replicates were treated as statistical units, whereas technical replicates and repeated instrumental measurements within the same preparation were averaged prior to analysis. For DPPH and ORAC assays, one IC_50_ value was calculated for each independent experiment from the corresponding concentration–response relationship. For all endpoints expressed as IC_50_, lower IC_50_ values indicate stronger antioxidant or membrane-protective activity. For CUPRAC, one IC_50_ value was calculated for each independent experiment as described in Section 2.5.3. IC_50_ values were likewise calculated independently for each extract, membrane model, and oxidative stressor in the membrane-protection assays. Pairwise comparisons between leaf and fruit extracts were performed using two-sided Welch’s *t*-tests. To control for multiple endpoint testing, *p*-values were adjusted using the Holm procedure; adjusted values (*p*_adj_) < 0.05 were considered statistically significant. Literature-derived Trolox values were not included in statistical comparisons. For membrane-protection data involving both extract type and membrane model, factorial effects were additionally evaluated using two-way ANOVA. Concentration-dependent hemolysis, GSH, DPH anisotropy, and Laurdan generalized polarization (GP) responses were analyzed using repeated-measures models accounting for extract type and concentration, with Dunnett-adjusted post hoc comparisons against the corresponding vehicle control where appropriate. To obtain an integrated measure of the Laurdan response across the tested concentration range, the concentration–response profile was summarized as the area under the curve (AUC), calculated separately for each independent experiment and membrane model. Leaf and fruit AUC values were subsequently compared using Welch’s t-test with Holm adjustment. For cross-endpoint visualization, parameters were oriented so that higher values represented stronger antioxidant activity, membrane protection, or membrane response. Accordingly, DPPH, ORAC, membrane-protection IC_50_, and CUPRAC values were transformed to 1/IC_50_. For the integrated descriptive heatmap, each parameter was normalized independently to the higher of the two extract values, which was assigned a value of 1.0. Thus, heatmap values represent within-parameter relative patterns and were not used for inferential testing. Directional effect sizes were expressed as log_2_ (fruit/leaf) ratios of the correspondingly oriented endpoints, with positive values indicating a fruit-extract advantage and negative values indicating a leaf-extract advantage (Figure S4). Statistical evidence across endpoints was additionally summarized as signed −log_10_(*p*_adj_), with the sign determined by the direction of the fruit-to-leaf effect; non-significant comparisons were treated as zero in the statistical outcome matrix. Statistical significance was accepted at *p*_adj_ < 0.05. Analyses were performed using Statistica software 16.1 (Krakow, Poland).

## 3. Results and Discussion

### 3.1. Phytochemical Analyses

**Figure 1:**
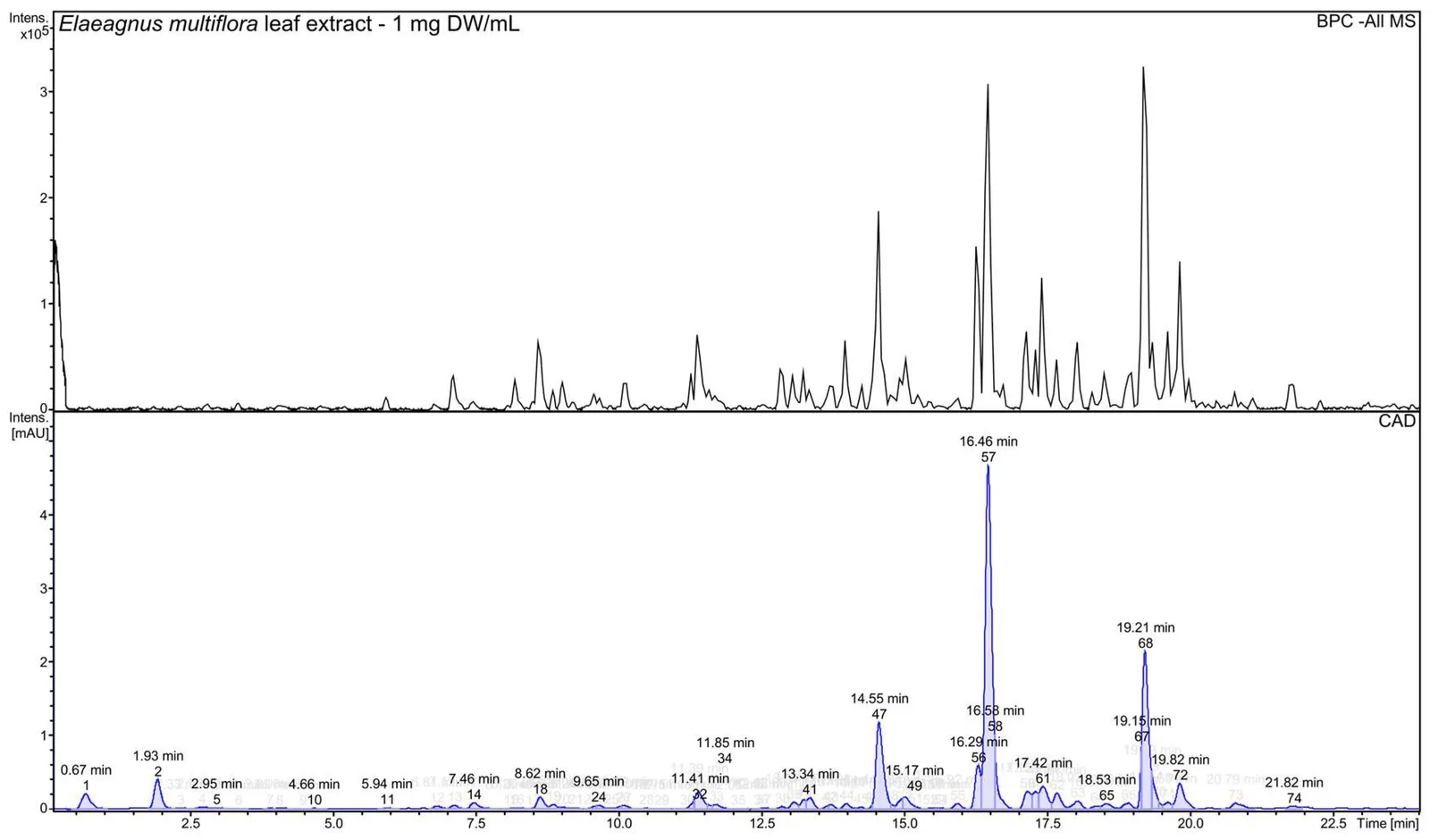
UHPLC-CAD-HR-QTOF-MS analysis of *Elaeagnus multiflora* leaf extract. Peak numbering corresponds to Table 1.

**Figure 2:**
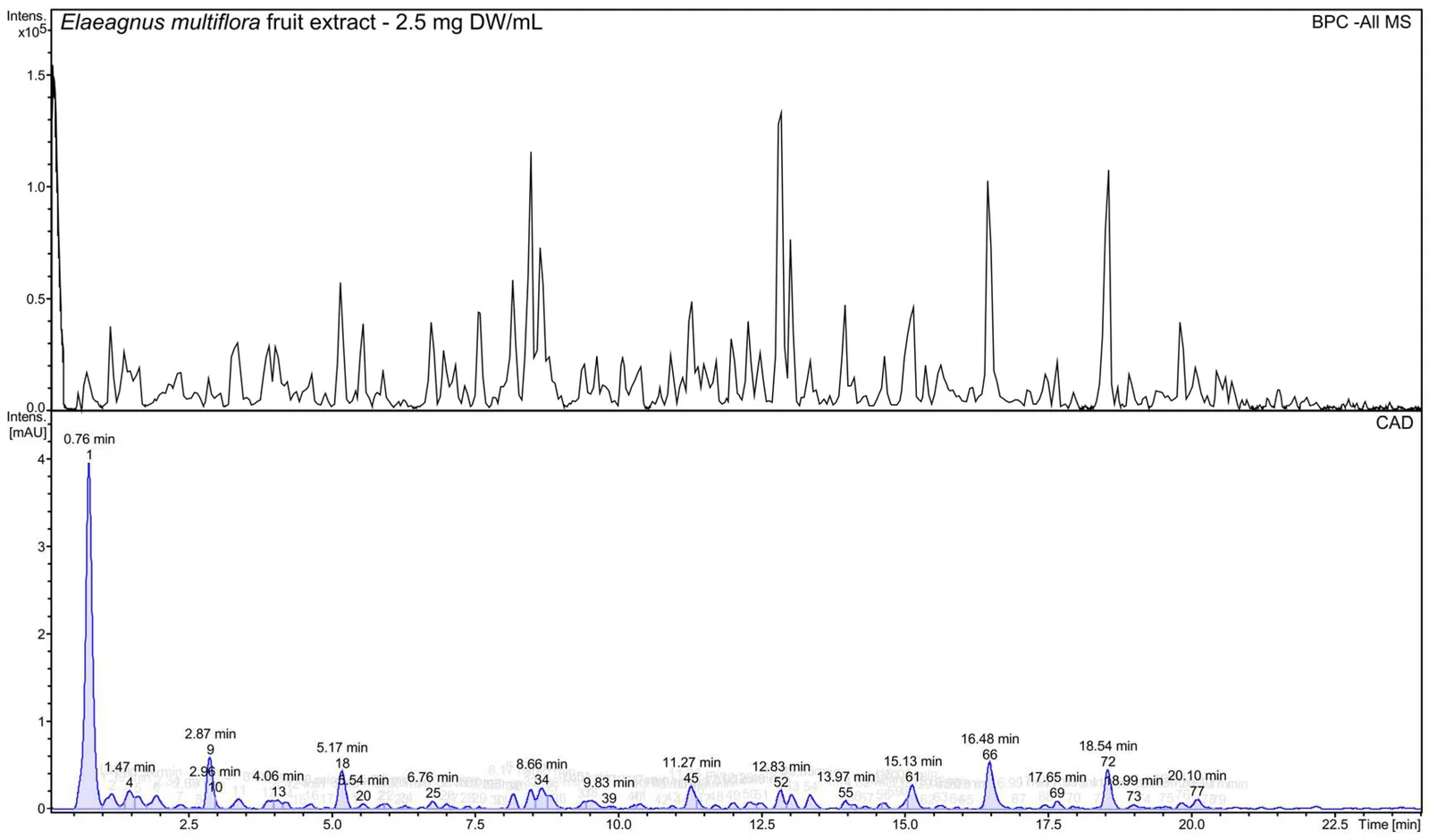
UHPLC-CAD-HR-QTOF-MS analysis of *Elaeagnus multiflora* fruit extract. Peak numbering corresponds to Table 2.

Comprehensive phytochemical profiling of *Elaeagnus multiflora* leaf and fruit extracts was performed using UHPLC–CAD–HR–QTOF–MS/MS, while the principal phenolic classes were quantified by UHPLC–UV. The datasets comprised 74 chromatographic peaks containing 119 annotated signals in the leaf extract and 79 peaks containing 135 annotated signals in the fruit extract (Figure 1 and Figure 2; Table 1 and Table 2). Compounds verified using authentic standards were distinguished from tentative annotations based on accurate mass, elemental composition, UV–Vis characteristics, MS/MS fragmentation, and molecular formula and structure candidates supported by SIRIUS/CSI:FingerID. Positional or stereochemical assignments not supported by authentic standards should therefore be interpreted with appropriate caution. Overall, the two plant organs exhibited clearly distinct qualitative and quantitative phytochemical profiles. Previous studies of *E. multiflora* have likewise demonstrated pronounced organ- and genotype-dependent variation in phytochemical composition, with leaves generally characterized by a particularly rich flavonol fraction and fruits showing a more heterogeneous phenolic profile [2,3,4,5,29]. The substantially broader number and structural diversity of signals detected in the present study extend these earlier targeted phytochemical characterizations.

The early region of the leaf chromatogram contained several flavan-3-ol and proanthocyanidin-related constituents. A signal at *m*/*z* 913.180, with characteristic product ions at *m*/*z* 727.128, 609.123, 423.072, and 305.066, was assigned to a prodelphinidin trimer, while signals at *m*/*z* 609.12 were consistent with B-type prodelphinidin dimers. Additional signals were tentatively assigned to a mixed procyanidin/prodelphinidin dimer, (epi)gallocatechin, catechin, and an (epi)catechin triglycoside. These observations confirm that condensed flavan-3-ol structures constitute a distinct component of the leaf extract and complement previous reports of proanthocyanidins in *Elaeagnus* tissues [3,4,29].

The leaf extract also showed considerable diversity of nitrogen-containing metabolites and hydroxycinnamic acid derivatives. Coumaroylagmatine and feruloylagmatine were detected at several retention times together with a subaphyllin-like hydroxycinnamate amide and other amide- or polyamine-related metabolites. Additional signals were consistent with putative non-covalent feruloylagmatine-containing ESI clusters and were therefore interpreted cautiously rather than as independently confirmed covalent structures. The hydroxycinnamate fraction included 5-chlorogenic acid, coumaroylquinic acid, feruloylquinic acid isomers, 5-*O*-sinapoylquinic acid, feruloyl-hexonic acid, and additional related conjugates. These results indicate that hydroxycinnamic acid derivatives and their nitrogen-containing conjugates represent a broader component of the leaf phytochemical profile than has generally been emphasized in previous targeted studies.

Despite this chemical diversity, flavonol glycosides clearly dominated the leaf extract. Their annotation was supported by UV spectra characteristic of flavonols and by aglycone-related product ions corresponding to kaempferol, quercetin, myricetin, and isorhamnetin derivatives. Numerous mono-, di-, tri-, and acylated glycosides were detected, with kaempferol and quercetin derivatives predominating. Quercetin 3-*O*-neohesperidoside and quercetin 3-*O*-glucoside were confirmed using authentic standards, whereas other glycosides were assigned tentatively according to their spectrometric characteristics. Consistent with previous studies of *E. multiflora* [3,4], kaempferol derivatives were especially prominent, including robinin- and lespedin-like compounds together with pentosylated, deoxyhexosylated, and hydroxycinnamoylated glycosides. The predominance of kaempferol- and quercetin-derived glycosides agrees with previous chromatographic studies of *E. multiflora* leaves, in which flavonols constituted the principal polyphenolic fraction and kaempferol derivatives were among the major compounds [3,4,5]. The present profile, however, reveals considerably greater structural diversity, including numerous differently glycosylated and acylated derivatives.

Several lower-abundance specialized metabolites were additionally detected in the leaf extract, including megastigmane- and norisoprenoid-related glycosides, iridoid- or monoterpene-related glycosides, methoxyphenolic glycosides, and phenylpropanoid- or (neo)lignan-related constituents. Signals tentatively assigned to phloretin di-*C*-hexoside and a flavanone/dihydrochalcone di-*C*-hexoside were also observed. Although many of these constituents occurred at relatively low chromatographic abundance and could not be quantified, their occurrence demonstrates that the leaf extract represents a chemically complex matrix in which dominant flavonol glycosides coexist with several other classes of specialized metabolites. The occurrence of norisoprenoid- and other isoprenoid-related constituents is also consistent with previous profiling of *E. multiflora* tissues [3,4,5], whereas the broader range of nitrogen-containing and amide-related signals observed here extends the chemical space previously described for this species.

Quantitative analysis confirmed the flavonol-rich character of the leaf extract. Peaks containing flavonol glycosides accounted for approximately 85.7% of the total integrated CAD signal. Because CAD response factors may differ among chemically distinct analytes and several chromatographic peaks contained coeluting constituents, these percentages represent relative contributions to the integrated CAD signal and should not be interpreted as direct mass or molar composition. The most intense chromatographic signals included peak 57 (32.16% of CAD area), peak 68 (12.57%), and peak 47 (9.08%). UHPLC–UV analysis yielded a total quantified phenolic content of 383.92 mg/g d.m., comprising 333.48 ± 1.80 mg rutin eq./g of flavonols, 30.14 ± 0.74 mg catechin eq./g of flavan-3-ols, and 20.18 ± 0.25 mg chlorogenic acid eq./g of hydroxycinnamic acid derivatives. Among the major individually quantified constituents were kaempferol *O*-deoxyhexosyl-hexoside *O*-deoxyhexoside (138.41 ± 1.22 mg/g), kaempferol 3,7-di-*O*-deoxyhexoside (61.80 ± 0.21 mg/g), and kaempferol *O*-pentosyl-hexoside *O*-deoxyhexoside (37.89 ± 0.05 mg/g). The colorimetrically determined total phenolic and condensed tannin contents were 255.20 ± 6.24 mg GAE/g d.m. and 74.78 ± 0.29 mg CE/g d.m., respectively.

In contrast, the fruit extract exhibited a substantially more heterogeneous chromatographic profile, comprising carbohydrate-derived constituents, phenolics, amino acid-related metabolites, and numerous glycosylated specialized metabolites. The earliest chromatographic region was dominated by carbohydrate-rich signals, with peak 1 accounting for 27.07% of the integrated CAD area. Additional early-eluting constituents included guanosine, phenylalanine, tryptophan, tryptophan pentoside, benzoxazinone-related compounds, and other nitrogen-containing metabolites. Phenylalanine and tryptophan were among the more intense individual CAD responses, representing 3.76% and 3.41% of the total integrated area, respectively.

Hydrolysable tannins and gallic acid derivatives represented one of the principal phenolic groups in the fruit extract. The profile included galloyl *O*-hexosides, methylgallate derivatives, gallic and methylgallic acids, and di-, tri-, tetra-, and pentagalloyl *O*-hexosides, together with signals tentatively assigned to terflavin B-, corilagin-, and casuarinin-like ellagitannins. Gallic acid and ellagic acid were confirmed using authentic standards. The total content of gallic acid derivatives reached 38.70 ± 0.23 mg gallic acid eq./g d.m. Among the quantitatively important constituents were tetragalloyl *O*-hexoside (6.20 ± 0.02 mg/g), digalloyl *O*-hexoside (5.05 ± 0.02 mg/g), corilagin-like derivatives, and pentagalloyl *O*-hexoside. These findings indicate that the fruit extract contains a structurally diverse galloylated fraction that extends beyond the phenolic groups emphasized in earlier targeted profiles of *E. multiflora* [3,4,27]. Hydrolysable tannins have previously been reported in the polyphenolic profile of *E. multiflora*, although their structural representation was considerably more limited than that observed here [3,4,5]. The present detection of multiple galloyl hexosides and ellagitannin-like constituents therefore indicates that the galloylated fraction of the fruit extract is substantially more complex than suggested by earlier targeted analyses.

Flavan-3-ols and proanthocyanidins constituted another major phenolic fraction in the fruit extract. Detected compounds included prodelphinidin trimers and B-type dimers, mixed procyanidin/prodelphinidin structures, (epi)gallocatechin isomers, catechin, and an (epi)catechin triglycoside. Total flavan-3-ols reached 36.84 ± 0.38 mg catechin eq./g d.m. Catechin, prodelphinidin trimer, and selected glycosylated or oligomeric flavan-3-ols were among the principal quantified constituents. The fruit extract therefore contained both condensed flavan-3-ol structures and an extensive hydrolysable tannin fraction.

Hydroxycinnamic acid derivatives in the fruit extract included 5-chlorogenic acid, coumaroylquinic acid, feruloylquinic acid, feruloyl-hexonic and -hexuronic acids, coumaroyl and sinapoyl *O*-hexosides, and other related conjugates. Their total content was 8.18 ± 0.06 mg chlorogenic acid eq./g d.m. Nitrogen-containing hydroxycinnamate conjugates, including *p*-coumaroylputrescine, feruloylagmatine isomers, and *N,N*-di-coumaroylspermidine-related compounds, further contributed to the structural diversity of this fraction.

Although flavonols represented a considerably smaller fraction of the fruit than of the leaf extract, several quercetin-, kaempferol-, myricetin-, isorhamnetin-, and gossypetin-related derivatives were detected. The fruit extract also contained megastigmane- and norisoprenoid-related glycosides, iridoid- or monoterpene-related compounds, and lignan- or phenylpropanoid-related constituents. Several of these occurred in coeluting chromatographic peaks; therefore, CAD-area percentages associated with such peaks cannot be assigned quantitatively to individual compounds.

Overall, UHPLC–UV quantified 90.34 mg/g d.m. of phenolic compounds in the fruit extract, with gallic acid derivatives and flavan-3-ols representing the largest quantified fractions. In contrast, the colorimetric assays yielded substantially higher responses, with total phenolic content of 490.22 ± 3.22 mg GAE/g d.m. and condensed tannin content of 855.91 ± 20.49 mg CE/g d.m. These colorimetric values are expressed as standard equivalents and should not be interpreted as directly equivalent to chromatographically determined mass fractions.

Taken together, the phytochemical analyses demonstrate a clear organ-dependent differentiation in *E. multiflora*. The leaf extract was characterized by a high abundance and structural diversity of flavonol glycosides, particularly kaempferol- and quercetin-derived compounds, together with prodelphinidins and hydroxycinnamic acid conjugates. The fruit extract exhibited a more heterogeneous phytochemical profile, with prominent contributions from galloylated hydrolysable tannins, flavan-3-ols, carbohydrate-derived constituents, amino acid-related metabolites, and norisoprenoid glycosides. These compositional differences provide the chemical basis for evaluating the distinct antioxidant responses of the two extracts in subsequent chemical and membrane-based assays. This organ-dependent differentiation is consistent with previous studies demonstrating substantial differences in polyphenolic and isoprenoid composition among *E. multiflora* fruits, seeds, and leaves [2,3,4,5]. However, the present high-resolution profiling reveals a broader chemical diversity, particularly within the galloylated, flavan-3-ol, hydroxycinnamate-conjugate, and nitrogen-containing fractions.

### 3.2. Antioxidant Activity Assays

The antioxidant activity of *Elaeagnus multiflora* fruit and leaf extracts was evaluated using three complementary assays representing different reaction mechanisms: DPPH, CUPRAC, and ORAC. DPPH reflects mixed hydrogen atom transfer (HAT) and single-electron transfer (SET) processes, CUPRAC primarily measures electron-donating/reducing capacity, whereas ORAC evaluates protection against peroxyl radical-induced oxidation predominantly through a HAT mechanism [30,31,32,33,34,35]. Accordingly, differences among these assays should be interpreted as complementary aspects of antioxidant behavior rather than as interchangeable measures of a single antioxidant property.

The two extracts exhibited clearly assay-dependent antioxidant profiles (Table 3). The fruit extract showed greater DPPH radical scavenging activity than the leaf extract, with IC_50_ values of 12.7 ± 0.7 and 63.5 ± 5.4 µg/mL, respectively. This difference was statistically significant after adjustment for multiple comparisons (*p*_adj_ = 0.003). The fruit extract also exhibited greater reducing capacity in the CUPRAC assay, with values of 11.4 ± 2.0 µg/mL compared with 33.1 ± 1.2 µg/mL for the leaf extract (*p*_adj_ < 0.001). In contrast, the ranking was reversed in the ORAC assay: the leaf extract showed the stronger response, with an IC_50_ of 2.2 ± 0.3 µg/mL compared with 6.0 ± 0.7 µg/mL for the fruit extract (*p*_adj_ = 0.004). Thus, neither plant organ exhibited a uniformly superior antioxidant response across all three chemical assays.

The contrasting antioxidant behavior corresponds with the marked differences in phytochemical composition observed between the two organs. The fruit extract contained substantial amounts of gallic acid derivatives, flavan-3-ols, and hydrolysable tannins, including multiple galloylated constituents. Such compounds possess multiple hydroxyl groups capable of participating in electron-transfer and radical scavenging reactions and may therefore contribute to the comparatively strong DPPH and CUPRAC responses of the fruit extract [32,36,37]. These associations should, however, be regarded as compositional relationships rather than evidence that individual compounds or compound classes were directly responsible for the observed activity.

The leaf extract displayed a different antioxidant pattern. Despite its weaker DPPH and CUPRAC responses, it exhibited the greater ORAC activity of the two extracts. Its phytochemical profile was dominated by kaempferol- and quercetin-derived glycosides and contained substantially higher amounts of UHPLC–UV-quantified flavonols and hydroxycinnamic acid derivatives than the fruit extract. Flavonols can participate effectively in peroxyl radical scavenging reactions, although their antioxidant behavior is strongly influenced by substitution pattern and glycosylation [38,39,40,41,42,43]. The enhanced ORAC response of the leaf extract is therefore consistent with its flavonol-rich composition, but the present data do not establish a direct causal contribution of individual glycosides.

Comparable relationships between organ-specific phytochemical composition and antioxidant performance have also been reported for other members of the *Elaeagnaceae* family. Studies of *Elaeagnus × submacrophylla*, *E. angustifolia*, and sea buckthorn have demonstrated substantial variation in phenolic and flavonoid profiles between plant organs and extracts, accompanied by differences in antioxidant responses depending on the assay and phytochemical matrix [42,43,44,45,46,47,48]. These observations further support the interpretation that antioxidant performance reflects both phytochemical composition and the analytical system used.

Comparison with the reference antioxidants further illustrates the assay dependence of antioxidant performance. In the DPPH assay, the fruit extract showed an IC_50_ similar to that of kaempferol, whereas in the CUPRAC assay its response was intermediate between kaempferol and Trolox. In ORAC, the leaf extract showed a lower IC_50_ than Trolox but remained less active than kaempferol. These comparisons demonstrate that the relative ranking of extracts and reference antioxidants changes according to the experimental system and therefore should not be generalized beyond the specific assay conditions used.

Overall, the chemical antioxidant assays revealed a clear divergence between the two plant organs: the fruit extract was favored in DPPH and CUPRAC, whereas the leaf extract was favored in ORAC. This pattern supports the use of complementary antioxidant assays for complex phytochemical matrices and demonstrates that antioxidant performance cannot be adequately characterized using a single chemical endpoint [33,42]. Importantly, these assay-specific differences provide a basis for examining whether chemical antioxidant capacity predicts protective efficacy in the erythrocyte-derived membrane models evaluated in the following section.

### 3.3. Antioxidant Activity in Erythrocyte Membranes and Liposomal Systems

Although DPPH, CUPRAC, and ORAC assays provide complementary information on the radical scavenging and reducing properties of the extracts, chemical assays do not fully reproduce the physicochemical environment of biological membranes. Therefore, the protective effects of *Elaeagnus multiflora* fruit and leaf extracts were further evaluated using two erythrocyte-derived membrane models: erythrocyte ghost membranes (RBC ghosts), which retain a complex lipid–protein membrane architecture, and liposomes prepared from erythrocyte membrane lipids (RBCLs), representing the isolated lipid fraction. Oxidative stress was induced using either AAPH or UVC radiation, and IC_50_ values were determined from the corresponding oxidation kinetics recorded at different extract concentrations (Figures S1 and S2).

The IC_50_ values obtained for the two extracts are summarized in Table 4. The most pronounced difference was observed in the RBCL model under AAPH-induced oxidation. The fruit extract showed an IC_50_ of 3.8 ± 1.2 µg/mL, compared with 9.5 ± 1.1 µg/mL for the leaf extract, corresponding to significantly greater protection by the fruit extract (*p*_adj_ = 0.016). Thus, under these experimental conditions, the fruit extract was more effective in protecting the isolated erythrocyte lipid fraction against peroxyl radical-mediated oxidation.

In contrast, no significant differences between the extracts were observed in the remaining membrane–stressor combinations. Under AAPH-induced oxidation of RBC ghosts, the IC_50_ values were 18.9 ± 2.2 µg/mL for the fruit extract and 19.7 ± 2.6 µg/mL for the leaf extract (*p*_adj_ = 0.732). Under UVC-induced oxidation, the corresponding values were 14.8 ± 4.7 and 14.9 ± 1.0 µg/mL in RBC ghosts and 21.2 ± 5.1 and 23.0 ± 6.4 µg/mL in RBCL liposomes for the fruit and leaf extracts, respectively (*p*_adj_ = 0.927 for both comparisons). Consequently, the advantage of the fruit extract was specific to AAPH-induced oxidation in the RBCL model rather than being a general feature across all membrane systems.

Membrane lipid peroxidation is an important consequence of oxidative stress in erythrocytes and can alter membrane organization, permeability, and mechanical properties, ultimately compromising membrane integrity [13]. The RBCL model, therefore, enables antioxidant protection of the erythrocyte lipid fraction to be examined independently of membrane proteins. In RBC ghosts, however, oxidative damage may additionally involve membrane-associated and cytoskeletal proteins, including spectrin, ankyrin, and band 3 protein [49,50]. The structural differences between these two models provide an important context for the observed model-dependent antioxidant responses and indicate that efficacy in an isolated lipid system cannot necessarily be extrapolated directly to a more complex lipid–protein membrane.

For contextual comparison, Bonarska-Kujawa et al. [9] reported Trolox IC_50_ values of 14.6 ± 1.3 µg/mL under UVC-induced oxidation and 3.9 ± 0.3 µg/mL under AAPH-induced oxidation of porcine erythrocyte ghost membranes. The IC_50_ values obtained for the fruit and leaf extracts in human RBC ghosts exposed to UVC radiation were therefore numerically close to the literature-derived Trolox value. Under AAPH-induced oxidation of RBC ghosts, both extracts required higher concentrations than the corresponding literature-derived Trolox reference to achieve 50% inhibition. However, because the Trolox values were obtained using porcine erythrocyte membranes in an independent study and were not measured in parallel with the present human membrane preparations, these comparisons are provided only as contextual reference data and were not included in the statistical analysis [9]. Moreover, no corresponding Trolox data are available for the RBCL model; therefore, no comparison with Trolox was made for the RBCL results.

Importantly, the membrane-based results did not reproduce the ranking obtained in the chemical antioxidant assays. The leaf extract exhibited the strongest ORAC response, yet this did not translate into superior protection in either RBC ghosts or RBCL liposomes. Conversely, the fruit extract showed a selective advantage in AAPH-treated RBCL despite the absence of a consistent advantage across the remaining membrane conditions. This divergence indicates that antioxidant activity measured in homogeneous chemical systems does not alone determine protective efficacy in membrane environments.

The divergence between the stronger ORAC response of the leaf fraction and its lack of superior membrane protection may partly reflect the different physicochemical environments of these assays. ORAC evaluates peroxyl radical scavenging in a predominantly aqueous system, whereas protection of erythrocyte-derived membranes additionally depends on the ability of antioxidant constituents to approach, partition into, or interact with the lipid–water interfacial environment. The predominance of glycosylated flavonols in the leaf fraction may favor antioxidant performance in the aqueous ORAC system without necessarily conferring greater efficacy at membrane interfaces. Conversely, the composition of the fruit fraction, including galloylated constituents and flavan-3-ols, may contribute to its selective activity in the lipid membrane model. These relationships remain mechanistic interpretations rather than evidence of direct localization of individual compounds within the membrane.

The results demonstrate that the antioxidant performance of *E. multiflora* extracts depends on both the oxidative stressor and the membrane model. The fruit extract showed a selective advantage against AAPH-induced oxidation of the isolated erythrocyte lipid fraction, whereas the two extracts displayed comparable protection in RBC ghosts and under UVC-induced oxidation. These findings support the complementary use of chemical and membrane-based models when evaluating antioxidant activity of chemically complex plant extracts.

### 3.4. Interaction of Extracts with Erythrocytes

**Figure 3:**
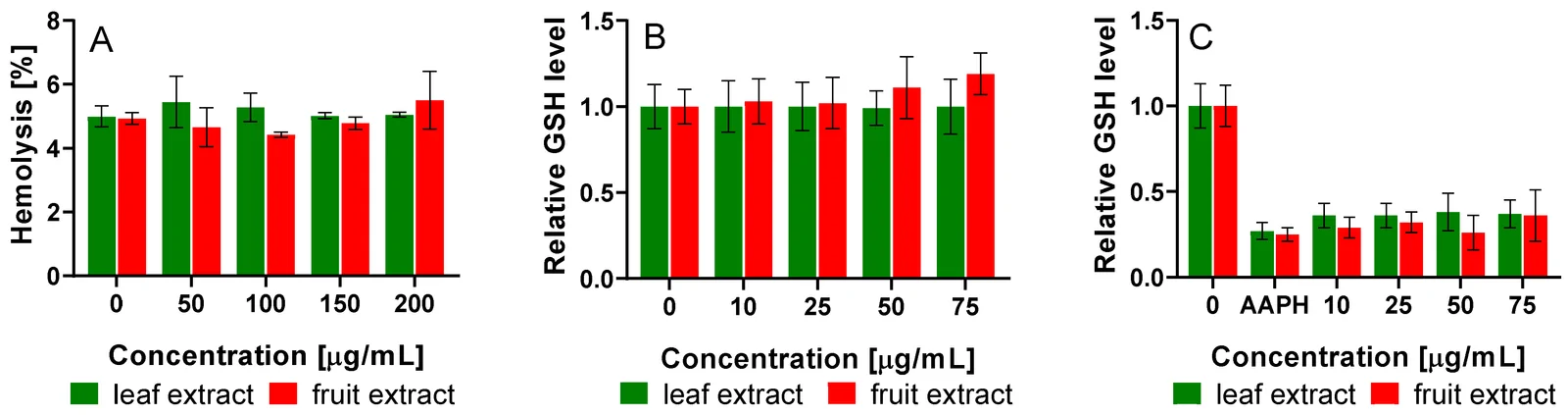
The percentage of hemolysis of erythrocytes modified with 50–200 µg/mL *Elaeagnus multiflora* extracts versus NaCl concentration (**A**). Concentration of reduced glutathione (GSH) in red blood cells modified with *Elaeagnus multiflora* Thunb. extracts (**B**). Concentration of reduced glutathione (GSH) in red blood cells modified with *Elaeagnus multiflora* Thunb. extracts and exposed to the oxidizing agent AAPH (**C**).

The hemolysis assay showed that neither the fruit nor the leaf extract increased erythrocyte hemolysis relative to the corresponding control over the tested concentration range of up to 200 µg/mL (Figure 3A). Thus, under the applied experimental conditions and exposure time, both extracts were compatible with erythrocyte membrane integrity.

Neither extract significantly affected intracellular reduced glutathione (GSH) levels under basal conditions (Figure 3B). Following AAPH-induced oxidative stress, GSH levels decreased relative to the non-oxidized control, and treatment with either extract did not significantly prevent this depletion (Figure 3C). These findings indicate that the membrane-protective effects observed in the RBC ghost and RBCL models were not accompanied by preservation of the intracellular glutathione pool. Because erythrocyte redox homeostasis involves both intracellular glutathione metabolism and the handling of oxidized glutathione species, the absence of a measurable effect on GSH suggests that the protective activity of the extracts occurred independently of a detectable modulation of this intracellular antioxidant system [51,52].

The different phytochemical compositions of the leaf and fruit extracts may nevertheless be relevant to their distinct biological responses. The leaf extract was dominated by kaempferol glycosides, whereas the fruit extract contained higher proportions of hydrolysable tannins, gallic acid derivatives, and flavan-3-ols. Kaempferol and its glycosylated derivatives have previously been shown to influence cellular antioxidant status and glutathione-related responses, although their biological activity depends strongly on chemical structure and glycosylation [51]. In the present erythrocyte model, however, the flavonol-rich leaf extract did not significantly preserve GSH during AAPH-induced oxidative stress, indicating that its high ORAC activity did not translate into measurable protection of the intracellular glutathione pool.

Taken together, the hemolysis and GSH results show that the effects of *E. multiflora* extracts were more evident at the membrane level than in the intracellular GSH response. The lack of increased hemolysis further supports short-term in vitro hemocompatibility of both extracts at the concentrations tested, whereas the absence of GSH preservation indicates that their membrane-protective effects are unlikely to be primarily mediated through maintenance of intracellular glutathione levels.

### 3.5. The Impact of Extract Components on the Hydrophilic and Hydrophobic Regions of Erythrocyte Membranes

**Figure 4:**
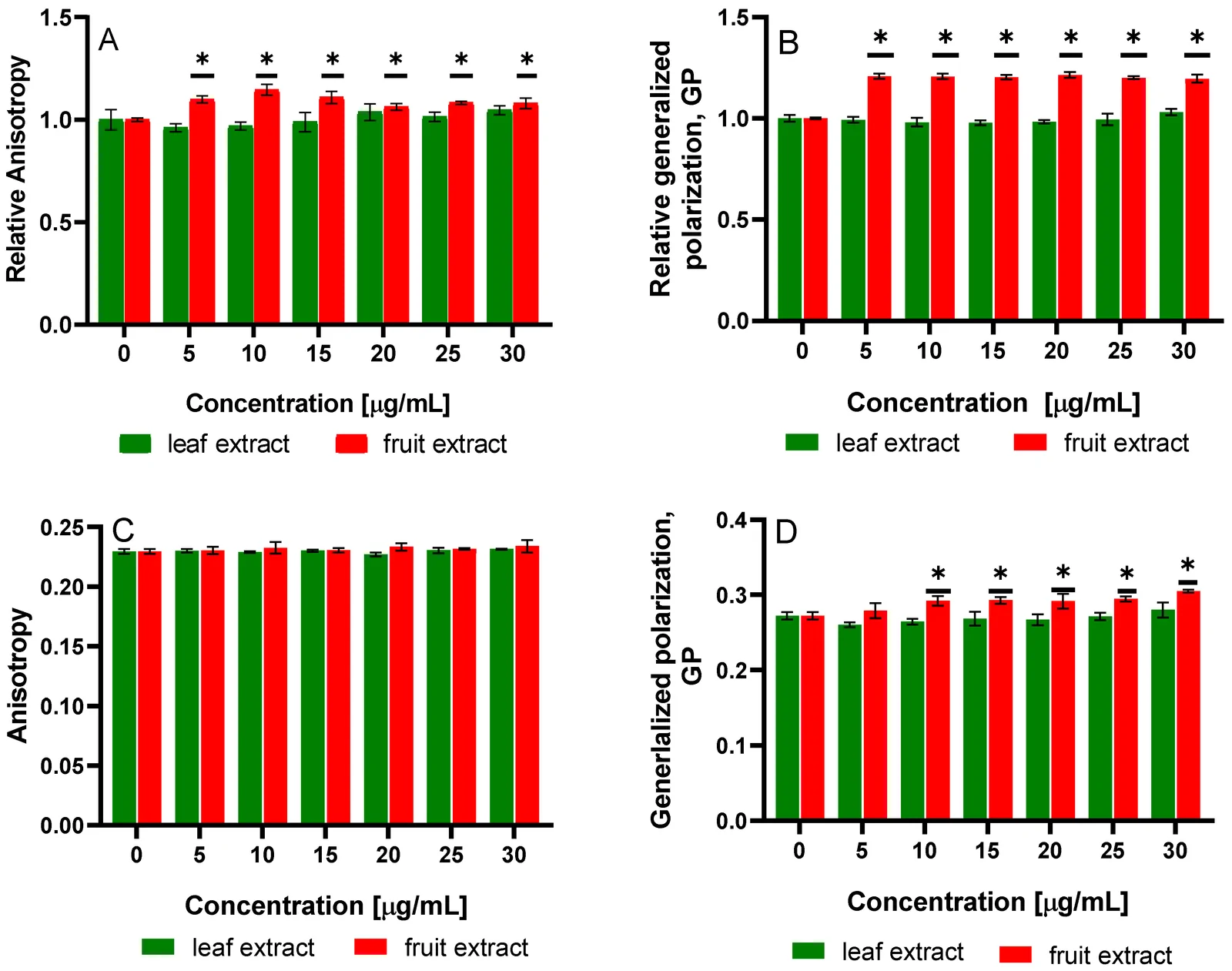
Relative values of DPH anisotropy (**A**) and Laurdan generalized polarization (GP) (**B**) measured in erythrocyte membranes after treatment with different concentrations (5.0–30.0 µg/mL) of *Elaeagnus multiflora* Thunb. leaf and fruit extracts. Values of DPH anisotropy (**C**) and Laurdan GP (**D**) measured in model membranes prepared from erythrocyte lipids after treatment with the same extract concentrations. Measurements were performed at 37 °C. * Statistically significant differences compared with the control are denoted at α < 0.05.

The effects of *Elaeagnus multiflora* extracts on membrane organization were evaluated using DPH and Laurdan fluorescence probes (Figure 4). DPH reports changes in the hydrophobic acyl-chain region of the lipid bilayer [53]. The leaf extract did not significantly alter DPH fluorescence anisotropy over the tested concentration range (5–30 µg/mL), indicating no measurable effect on the fluidity of the hydrophobic membrane region under the applied conditions. In contrast, the fruit extract produced a small but statistically significant increase in DPH anisotropy in erythrocyte ghost membranes (Figure 4A). The magnitude of this response remained relatively similar across the concentration range and therefore did not show a clear dose-dependent pattern.

Notably, the DPH response observed for the fruit extract in erythrocyte ghost membranes was not reproduced in the protein-free RBCL model, where DPH anisotropy remained essentially unchanged (Figure 4C). Erythrocyte membranes retain a complex architecture comprising lipids, integral and peripheral proteins, membrane asymmetry, cholesterol-rich regions, and cytoskeleton-associated domains [54]. This structural complexity may therefore contribute to the different fluorescence responses observed between RBC ghosts and lipid-only membranes. Tannins and other galloylated polyphenols can influence membrane-associated systems, and the magnitude and direction of these effects depend strongly on molecular structure and the number and position of galloyl groups [55,56]. Accordingly, the modest DPH response observed for the fruit extract is consistent with an alteration of the local physicochemical environment sensed by the probe, but it does not by itself demonstrate direct binding to membrane proteins or deeper incorporation of individual fruit-derived constituents into the bilayer.

A clearer differentiation between the extracts was observed with Laurdan. Laurdan generalized polarization (GP) is sensitive to changes in polarity, hydration, and lipid packing in the interfacial region of membranes [28,57]. The leaf extract produced no significant changes in Laurdan-derived parameters, whereas the fruit extract altered GP in both erythrocyte ghost membranes and RBCL liposomes (Figure 4B,D). When the concentration-dependent Laurdan responses were integrated as area under the curve (AUC), the fruit extract showed a significantly greater overall GP response than the leaf extract in both RBC ghosts (*p*_adj_ < 0.001) and RBCL liposomes (*p*_adj_ = 0.020) (Figure S6). The difference was more pronounced in RBC ghosts than in the lipid-only model.

These findings indicate that constituents of the fruit extract affected the physicochemical environment of the membrane interfacial region more strongly than constituents of the leaf extract under the conditions examined. Importantly, changes in Laurdan GP reflect alterations in the local hydration and packing environment sensed by the probe and should not be interpreted as direct measurements of compound localization, membrane penetration depth, or binding affinity. Nevertheless, the response in both RBC ghosts and RBCL liposomes suggests that the effect was not restricted to the protein-containing membrane system and included changes detectable within the lipid interfacial environment.

The phytochemical differences between the extracts provide a plausible context for these contrasting responses. The fruit extract contained a substantial proportion of hydrolysable tannins, gallic acid derivatives, and flavan-3-ols. Polyphenol–lipid interactions are strongly dependent on molecular structure, hydrophobicity, the number of galloyl groups, and membrane composition [58]. The greater Laurdan response of the fruit extract is therefore consistent with the presence of structurally diverse galloylated constituents, although the present experiments cannot identify which individual compounds were responsible for the observed membrane response.

In contrast, the leaf extract was dominated by kaempferol and quercetin glycosides and produced no measurable changes in either DPH anisotropy or Laurdan GP. Glycosylation increases the hydrophilic character of flavonoids and can substantially modify their interaction with lipid membranes compared with the corresponding aglycones [59]. This provides a possible explanation for the comparatively limited membrane response of the leaf extract, but the absence of fluorescence changes does not establish that its constituents were restricted to the membrane surface or excluded from the bilayer. The absence or small magnitude of changes in some fluorescence endpoints should also be interpreted cautiously, because the limited dynamic range of the probe response under the applied conditions may have reduced sensitivity to subtle additional alterations in membrane organization.

Overall, the fluorescence measurements revealed a clear organ-dependent difference in membrane response. The fruit extract affected the Laurdan-sensitive interfacial region in both membrane systems and produced a modest DPH response specifically in RBC ghosts, whereas the leaf extract did not cause measurable changes in either fluorescence parameter. Together with the antioxidant results, these findings indicate that the high ORAC activity of the leaf extract did not translate into a pronounced alteration of membrane organization, while the fruit extract combined stronger DPPH/CUPRAC activity and selective RBCL–AAPH protection with a more pronounced membrane-associated fluorescence response. Thus, antioxidant efficacy in these models appears to depend on the experimental context and cannot be predicted solely from chemical radical scavenging capacity.

### 3.6. Integrated Statistical Analysis of Antioxidant and Membrane Responses

**Figure 5:**
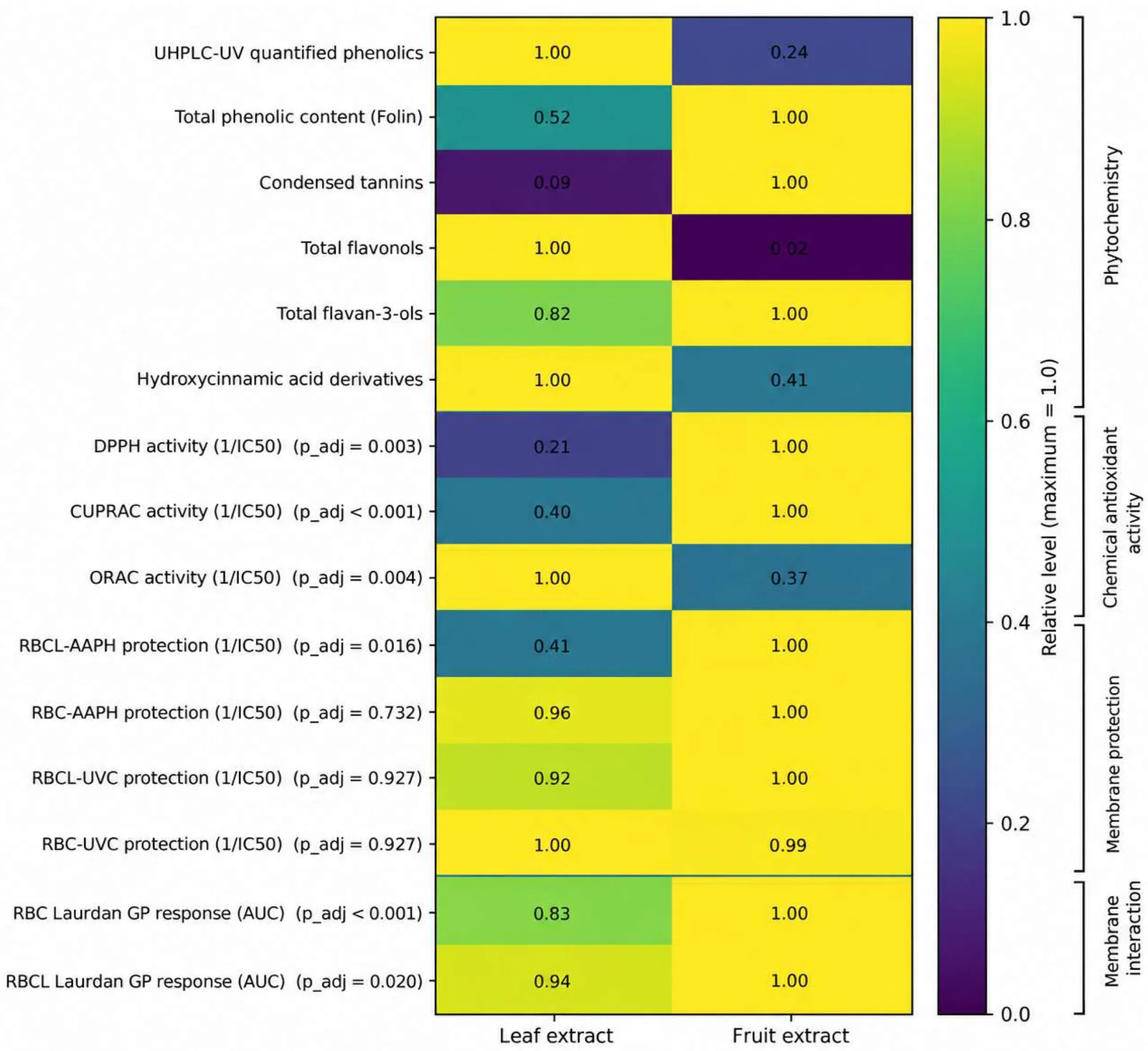
Integrated descriptive heatmap of phytochemical composition and antioxidant- and membrane-related responses of *Elaeagnus multiflora* Thunb. leaf and fruit extracts. For each parameter, values were oriented so that higher values represented a greater phytochemical contribution, antioxidant activity, membrane protection, or membrane response. DPPH, CUPRAC, ORAC and membrane-protection endpoints are expressed as 1/IC_50_. Each parameter was normalized independently to the higher value observed for the two extracts, which was assigned a value of 1.0; therefore, the displayed values represent relative within-parameter patterns and should not be interpreted as absolute effect sizes or used for comparisons between different rows. Laurdan GP responses were summarized as areas under the concentration–response curves (AUC). Adjusted *p*-values (*p*_adj_) shown for functional endpoints were obtained from fruit–leaf comparisons following Holm correction for multiple testing. The heatmap is intended as an integrated descriptive visualization and was not itself used for inferential statistical testing.

Integrated analysis across the experimental endpoints revealed no uniform ranking of the two *E. multiflora* extracts (Figure 5). Instead, the direction and magnitude of the fruit-to-leaf differences varied according to the assay and membrane model. After correction for multiple comparisons, the chemical assays separated the extracts in opposite directions, whereas most membrane-protection endpoints showed no significant difference (Figure S5). The only significant separation in the oxidation models occurred under AAPH-induced oxidation of RBCL, favoring the fruit extract.

Factorial analysis of the AAPH membrane-protection data confirmed significant effects of extract type (F_1,8_ = 8.53, *p* = 0.0193, partial η^2^ = 0.516) and membrane model (F_1,8_ = 137.95, *p* < 0.00001, partial η^2^ = 0.945). The extract × membrane model interaction approached, but did not reach, statistical significance (F_1,8_ = 5.10, *p* = 0.0539), indicating that the apparent amplification of the fruit–leaf difference in the lipid-only system should be interpreted cautiously.

The fluorescence-based membrane measurements provided an additional level of discrimination. Integrated Laurdan GP responses were greater for the fruit extract in both membrane systems, with the largest difference observed in erythrocyte ghost membranes. This pattern indicates a stronger fruit-associated alteration of the Laurdan-sensitive interfacial membrane environment, without implying direct localization or binding of individual phytochemicals. Importantly, neither extract increased erythrocyte hemolysis relative to the corresponding control over the tested concentration range, indicating that the Laurdan-detectable membrane responses were not accompanied by measurable loss of erythrocyte membrane integrity.

Taken together, the integrated dataset identifies two distinct functional profiles rather than a single hierarchy of antioxidant potency. The flavonol-rich leaf extract was characterized by a preferential ORAC response, whereas the fruit extract, enriched in galloylated compounds, hydrolysable tannins, and flavan-3-ols, was associated with stronger electron-transfer/radical scavenging responses, selective protection of the lipid membrane model under AAPH stress, and a more pronounced Laurdan-detectable membrane response. Chemical antioxidant activity did not consistently predict membrane-protective efficacy across the experimental models. The stronger DPPH and CUPRAC responses of the fruit extract co-varied with its greater protection in AAPH-treated RBCL liposomes, whereas this relationship was absent in the remaining membrane–stressor combinations and the superior ORAC response of the leaf extract did not translate into greater membrane protection.

## 4. Limitations

The present study was based on two independently prepared extracts per plant organ; therefore, the findings should be interpreted as characteristic of the investigated cultivar, harvest, and extraction conditions rather than as universally representative of *E. multiflora* fruits and leaves.

## 5. Conclusions

This study demonstrates that the antioxidant potential of *Elaeagnus multiflora* Thunb. cannot be described by a single measure of antioxidant capacity but rather arises from the interplay among organ-specific phytochemical composition, reaction mechanisms, and the membrane environment. The leaf and fruit extracts exhibited markedly different chemical profiles and, correspondingly, distinct functional signatures. The flavonol-rich leaf extract was preferentially effective in the ORAC system, whereas the fruit extract, characterized by a greater contribution of galloylated compounds, hydrolysable tannins, and flavan-3-ols, showed stronger activity in DPPH and CUPRAC assays and selective protection against AAPH-induced oxidation in erythrocyte lipid liposomes.

Importantly, antioxidant performance in chemical assays did not consistently predict membrane-protective efficacy across the erythrocyte-derived experimental systems. The stronger DPPH and CUPRAC responses of the fruit fraction co-varied with its greater protection against AAPH-induced oxidation in RBCL liposomes; however, this relationship was not reproduced in the remaining membrane–stressor combinations, and the stronger ORAC response of the leaf fraction did not translate into greater membrane protection. The stronger Laurdan response produced by the fruit extract further indicated a greater alteration of the physicochemical environment of the membrane interfacial region, while most other membrane-protection endpoints did not distinguish the two extracts. Neither extract increased erythrocyte hemolysis within the tested concentration range, whereas neither prevented AAPH-induced depletion of intracellular GSH. These findings indicate that the observed protective effects were expressed predominantly at the membrane-response level rather than through measurable preservation of the intracellular glutathione pool.

Overall, under the conditions investigated, the leaf- and fruit-derived phenolic-rich fractions of *E. multiflora* cv. ‘Marianna’ exhibited distinct phytochemical and functional profiles, emphasizing the importance of evaluating their biological activity using complementary chemical and membrane-based models. The organ-dependent profiles identified here provide a rational basis for selecting *E. multiflora*-derived phytochemical fractions according to the intended functional context rather than relying exclusively on total phenolic content or a single antioxidant assay. Further studies should determine the contribution of individual phytochemical groups, assess their stability and bioaccessibility, and establish whether the membrane-related effects observed in vitro are retained in more complex cellular and physiological systems.

## Figures and Tables

**Table 1:** UHPLC-HR-QTOF-MS/MS characteristics and content of metabolites in *Elaeagnus multiflora* leaf extract.

No	RT (min)	UV λmax (nm)	Observed [M − H]^−^, *m*/*z*	Formula	Error (ppm)	MS/MS Fragments ^a^	Identity	CAD Area (%) ^b^	Content (mg/g d.m.) ^c^
1	0.67	– ^d^	371.1195 [+FA] ^e^/325.1135	C_13_H_24_O_12_	−0.1	205.0721, 163.0614, 265.0928, 191.0569, 235.0823, 143.0352	disaccharide (deoxyhexosyl-hexose)	1.824	NQ ^f^
		–	317.0565	C_18_H_10_N_2_O_4_	0.8	225.0060, 164.9853	*N*-containing phenolic (alkaloid-type metabolite)		NQ
2	1.93	278	913.1803	C_45_H_38_O_21_	0.5	305.0664, 423.0720, 609.1232, 727.1282, 243.0308, 177.0196	prodelphinidin trimer ((epi)gallocatechin trimer)	2.706	20.67 ± 0.65
3	2.35	277	609.1242	C_30_H_26_O_14_	1.4	423.0715, 305.0665, 177.0192, 255.0294, 441.0829	B-type prodelphinidin dimer ((epi)gallocatechin dimer)	0.057	2.83 ± 0.04
4	2.71	278	303.0992	C_15_H_16_N_2_O_5_	−1.9	259.1085, 215.1181, 186.0926, 241.0873	*N*-containing phenolic (amide derivative)	0.234	NQ
5	2.95	275, 300	320.1984	C_17_H_27_N_3_O_3_	−1.4	304.1675, 247.1074, 204.0666	*N*-containing metabolite (amide/polyamine derivative)	0.108	NQ
6	3.34	275	305.0664	C_15_H_14_O_7_	0.8	125.0233, 219.0654, 261.0759, 167.0341, 261.0749	(epi)gallocatechin	0.063	0.51 ± 0.05
7	3.89	–	323.1724	C_15_H_24_N_4_O_4_	0.2	134.0367, 305.1619, 149.0598	*N*-containing metabolite (amide/polyamine derivative)	0.100	NQ
8	4.12	278	609.1246	C_30_H_26_O_14_	0.6	423.0703, 305.0655, 177.0189, 255.0294, 441.0813	B-type prodelphinidin dimer ((epi)gallocatechin dimer)	0.192	0.30 ± 0.01
		280, 317	331.0691	C_14_H_20_O_9_	1.5	153.0169, 168.0432	methoxyphenolic glycoside (leonuriside-like)		0.37 ± 0.01
9	4.45	290, 315	320.1978	C_17_H_27_N_3_O_3_	0.6	304.1670, 247.1069, 204.0661	*N*-containing metabolite (amide/polyamine derivative)	0.041	NQ
10	4.65	277	593.1305	C_30_H_26_O_13_	−0.8	305.0663, 423.0716, 255.0289, 467.0974	procyanidin/prodelphinidin dimer ((epi)catechin + (epi)gallocatechin)	0.085	0.14 ± 0.00
11	5.94	278	335.1243	C_16_H_20_N_2_O_6_	1.6	203.0819	oxygenated *N*-containing phenolic	0.189	NQ
		280, 310	355.0675	C_15_H_16_O_10_	−0.2	209.0297, 191.0194, 147.0325	hydroxycinnamate derivative		0.30 ± 0.00
12	6.81	278, 310sh	627.3251 [+FA]/581.3206	C_29_H_42_N_8_O_5_	2.2	275.1503, 305.1597, 233.1281, 425.2178, 290.1370	non-covalent heterodimer of feruloylagmatine + coumaroylagmatine	0.288	
		278	305.0659	C_16_H_18_O_8_	−1.2	125.0241, 219.0662, 261.0749, 167.0357	(epi)gallocatechin		<LLOQ
13	7.12	309	275.1503	C_14_H_20_N_4_O_2_	−0.9	233.1289, 119.0500	coumaroylagmatine	0.352	0.38 ± 0.00
		309	337.0929	C_16_H_18_O_8_	0.1	191.0554, 163.0401, 119.0497, 319.1297	coumaroylquinic acid isomer		0.42 ± 0.00
14	7.45	278	289.0715	C_15_H_14_O_6_	0.9	245.0821	catechin# ^g^	0.710	4.52 ± 0.34
		280, 320sh	611.3305/657.3351 [+FA]	C_30_H_44_N_8_O_6_	−1.2	305.1606, 289.1296, 247.1059, 204.0660, 455.2280, 272.1027	non-covalent dimer of feruloylagmatine		0.20 ± 0.00
15	8.10	323, 295sh	353.088	C_16_H_18_O_9_	−0.4	191.0560, 161.0244	5-chlorogenic acid#	0.047	0.60 ± 0.01
16	8.21	–	417.1036	C_17_H_22_O_12_	0.9	152.0106, 241.0710	iridoid-/monoterpene *O*-hexoside-like	0.191	NQ
		280, 315	263.1396	C_14_H_20_N_2_O_3_	1.9	248.1166, 247.1084, 204.0662	hydroxycinnamate amide (subaphyllin-like)		1.22 ± 0.01
17	8.5	–	457.1718	C_21_H_30_O_11_	−0.6	277.1080, 189.1271	megastigmane glycoside (amygdalactone *O*-hexoside)	0.061	NQ
18	8.62	321, 295sh	305.1612	C_15_H_22_N_4_O_3_	−2.1	289.1303, 272.1039, 247.1084, 204.0665, 190.0504	feruloylagmatine	1.144	1.35 ± 0.01
19	8.85	255, 265, 353	757.1833	C_32_H_38_O_21_	−0.1	299.0195, 271.0244, 462.0804, 595.1294	quercetin *O*-pentosyl-hexoside *O*-hexoside	0.410	2.31 ± 0.02
20	9.00	323, 295sh	367.1028	C_17_H_20_O_9_	1.7	193.0505, 134.0364	feruloylquinic acid	0.136	1.67 ± 0.00
21	9.23	323, 295sh	367.1031	C_17_H_20_O_9_	1.0	193.0505, 134.0361	feruloylquinic acid	0.129	
22	9.47	–	439.1617	C_21_H_28_O_10_	−1.7	195.0653, 391.1395, 165.0560, 150.0318, 180.0434	(neo)lignan derivative	0.039	NQ
23	9.63	306	275.1509	C_14_H_20_N_4_O_2_	0.2	233.1291, 119.0504, 191.1193	coumaroylagmatine	0.295	1.11 ± 0.01
24	9.75	–	431.1194	C_18_H_24_O_12_	0.2	152.0106	iridoid *O*-hexoside (asperulosidic acid-like)	0.137	NQ
		266, 343	755.2032	C_33_H_40_O_20_	1.1	593.1468, 283.0240, 255.0292	kaempferol *O*-deoxyhexosyl-hexoside *O*-hexoside		0.37 ± 0.01
25	9.88	255, 265, 353	625.1406	C_27_H_30_O_17_	0.7	299.0195, 271.0242, 315.0148, 463.0870	quercetin di-*O*-hexoside	0.071	0.93 ± 0.03
26	10.09	265, 346	741.1886	C_32_H_38_O_20_	−0.4	283.0253, 579.1355, 255.0289, 227.0382	kaempferol *O*-pentosyl-hexoside *O*-hexoside	0.122	
		266, 343	901.2608	C_39_H_50_O_24_	1.3	283.0250, 739.2099, 255.0299, 227.0340, 446.0869	kaempferol *O*-deoxyhexosyl-deoxyhexosyl-hexoside *O*-hexoside		0.45 ± 0.01
27	10.12	324, 295sh	397.1144	C_18_H_22_O_10_	−1.0	223.0608, 149.0238, 164.0478	5-*O*-sinapoylquinic acid	0.261	1.62 ± 0.00
28	10.43	282, 325	371.0986	C_16_H_20_O_10_	−0.5	249.0620, 121.0281	feruloyl-hexonic acid	0.088	0.08 ± 0.00
		–	435.2231 [+FA]/389.2198	C_20_H_36_O_10_	1.0	295.0617	megastigmane *O*-hexoside		NQ
29	10.72	–	349.1880/395.1929 [+FA]	C_16_H_30_O_8_	−1.5	179.0541, 223.0619, 307.1150	megastigmane *O*-hexoside	0.092	NQ
		–	521.2604/567.2652 [+FA]	C_24_H_42_O_12_	0.2	389.2218, 293.0909, 227.1636, 155.1068	megastigmane *O*-pentosyl-hexoside		NQ
30	11.10	–	433.2086/479.2061 [+FA]	C_20_H_34_O_10_	−1.5	161.0430	oxygenated megastigmane *O*-hexoside	0.074	NQ
		283, 320sh	595.1699	C_27_H_32_O_15_	0.1	355.0822, 385.0940, 475.1256, 235.0242, 313.0723	flavanone/dihydrochalcone di-*C*-hexoside (naringenin-like)		NQ
31	11.26	–	401.1450/447.1511 [+FA]	C_18_H_26_O_10_	0.7	269.1016, 161.0458	oxygenated megastigmane *O*-pentoside	0.298	NQ
32	11.39	264, 345	609.1459	C_27_H_30_O_16_	1.7	283.0253, 255.0289, 227.0349	kaempferol *O*-hexosyl-hexoside	1.787	0.79 ± 0.01
		316, 295sh	305.1618	C_15_H_22_N_4_O_3_	0.5	289.1308, 272.1043, 247.1085, 204.0666, 190.0504	feruloylagmatine		8.46 ± 0.25
33	11.57	–	521.2605/567.2659 [+FA]	C_24_H_42_O_12_	0.2	389.2218, 293.0899, 227.1652, 155.1072	megastigmane *O*-pentosyl-hexoside	0.366	NQ
34	11.71	265, 343	755.2045	C_33_H_40_O_20_	−0.7	593.1512, 283.0246, 255.0296, 227.0350, 446.0847	kaempferol *O*-deoxyhexoside *O*-hexoside *O*-hexoside	0.565	1.47 ± 0.01
		–	401.1451/447.1505 [+FA]	C_18_H_26_O_10_	0.7	269.1031, 161.0458	oxygenated megastigmane *O*-pentoside		NQ
35	12.06	265, 346	579.1347	C_26_H_28_O_15_	1.5	283.0246, 255.0292, 446.0865, 417.0820	kaempferol *O*-hexoside *O*-pentoside	0.062	0.68 ± 0.08
		–	521.2607/567.2659 [+FA]	C_24_H_42_O_12_	0.2	389.2189, 293.0909, 227.1657, 155.1073	megastigmane *O*-pentosyl-hexoside		NQ
36	12.44	–	431.1921	C_20_H_32_O_10_	0.3	385.1865, 223.1332, 205.1252, 325.0935, 191.0285, 164.0476	oxygenated megastigmane *O*-hexoside	0.032	NQ
37	12.53	267, 335	609.1454	C_27_H_30_O_16_	1.2	299.0197, 271.0238, 255.0300, 447.0923, 463.0878	quercetin *O*-hexoside *O*-deoxyhexoside	0.156	0.36 ± 0.01
		–	517.2285/563.2341 [+FA]	C_24_H_38_O_12_	1.1	205.1236, 293.0859, 385.1845	megastigmane *O*-glycoside		NQ
38	12.86	273, 325	371.0985	C_16_H_20_O_10_	−0.3	249.0611, 121.0297, 353.0877	feruloyl-hexonic acid	0.260	0.09 ± 0.00
39	13.06	–	517.2285/563.2330 [+FA]	C_24_H_38_O_12_	1.1	205.1227, 293.0876, 233.0654	megastigmane *O*-glycoside	0.707	NQ
		265, 341	755.2045	C_33_H_40_O_20_	−0.7	609.1471, 283.0253, 255.0289, 227.0349, 430.0906	kaempferol *O*-deoxyhexosyl-hexoside *O*-hexoside		0.91 ± 0.02
40	13.24	254, 265, 351	741.1878	C_32_H_38_O_20_	0.7	299.0192, 271.0241, 446.0844, 595.1305	quercetin *O*-pentosyl-hexoside *O*-deoxyhexoside	0.734	6.24 ± 0.02
41	13.34	255, 265, 349	887.2455	C_38_H_48_O_24_	0.9	299.0191, 271.0240, 741.1882, 446.0849, 255.0289	quercetin *O*-pentosyl-deoxyhexosyl-hexoside *O*-deoxyhexoside	1.176	
		–	435.2227 [+FA]/389.2188	C_20_H_36_O_10_	2.0	295.0620, 227.1671, 310.0848, 283.0641	megastigmane *O*-hexoside (alangionoside A-type compound)		NQ
42	13.61	323, 295sh	563.1759	C_27_H_32_O_13_	2.0	193.0507, 389.1232, 150.0315, 341.1023, 165.0553	hydroxycinnamic acid derivative	0.191	0.54 ± 0.00
		254, 265, 351	741.1881	C_32_H_38_O_20_	0.5	299.0189, 271.0237, 446.0856, 593.1298	quercetin *O*-pentosyl-hexoside *O*-deoxyhexoside		1.51 ± 0.02
		265, 339	739.2086	C_33_H_40_O_19_	0.7	283.0241, 255.0291, 593.1516, 227.0346, 430.0879	kaempferol *O*-deoxyhexosyl-hexoside *O*-deoxyhexoside (robinin-like)		
43	13.71	–	415.1606/461.1656 [+FA]	C_19_H_28_O_10_	1.0	191.0553, 149.0444, 311.0984, 251.0762, 161.0449	oxygenated megastigmane *O*-hexoside	0.600	NQ
44	13.99	–	521.2594/567.2648 [+FA]	C_24_H_42_O_12_	1.9	389.2195, 227.1645	megastigmane *O*-pentosyl-hexoside	0.737	NQ
		268, 305	319.0449	C_15_H_12_O_8_	3.1	183.0300, 139.0380, 153.0194	hydroxycinnamic acid derivative		0.21 ± 0.01
45	14.25	–	521.2597/567.2647 [+FA]	C_24_H_42_O_12_	1.9	389.2175, 293.0877	megastigmane *O*-pentosyl-hexoside	0.348	NQ
46	14.39	–	415.1606/461.1656 [+FA]	C_19_H_28_O_10_	1.0	191.0553, 149.0444, 311.0984, 251.0762, 161.0449	oxygenated megastigmane *O*-hexoside	0.204	NQ
		266, 341	593.151	C_27_H_30_O_15_	0.4	283.0237, 255.0291, 447.0912, 227.0345	kaempferol *O*-hexoside *O*-deoxyhexoside		37.89 ± 0.05
47	14.55	265, 346	725.1922	C_32_H_38_O_19_	1.7	283.0240, 255.0294, 447.0928, 227.0346, 430.0901	kaempferol *O*-pentosyl-hexoside *O*-deoxyhexoside	9.080	
		265, 345	871.2497	C_38_H_48_O_23_	1.9	283.0241, 725.1926, 255.0293, 227.0343, 430.0893	kaempferol *O*-pentosyl-deoxyhexosyl-hexoside *O*-deoxyhexoside		0.42 ± 0.01
		254, 265, 351	741.1874	C_32_H_38_O_20_	1.3	271.0238, 300.0271, 255.0289, 243.0296	quercetin *O*-pentosyl-deoxyhexosyl-hexoside		
48	14.75	*–*	415.1607/461.1656 [+FA]	C_19_H_28_O_10_	0.8	191.0553, 149.0444, 311.0984, 251.0762, 161.0449	oxygenated megastigmane *O*-hexoside	0.311	NQ
49	14.87	255, 265, 349	609.1456	C_27_H_30_O_16_	0.8	299.0192, 271.0242, 446.0845, 255.0295	quercetin *O*-hexoside *O*-deoxyhexoside	0.970	2.97 ± 0.01
		266, 327	725.1924	C_32_H_38_O_19_	1.4	283.0241, 579.1345, 255.0293, 227.0347, 430.0886	kaempferol *O*-pentosyl-hexoside *O*-deoxyhexoside		
50	15.00	254, 349	609.1457	C_27_H_30_O_16_	0.6	315.0145, 287.0190, 462.0797	myricetin *O*-deoxyhexoside *O*-deoxyhexoside	1.119	2.39 ± 0.02
		255, 267, 351	755.2031	C_33_H_40_O_20_	1.2	299.0193, 271.0240, 609.1456, 446.0838	quercetin *O*-deoxyhexosyl-hexoside *O*-deoxyhexoside		
		266, 327	595.1296	C_26_H_28_O_16_	1.5	271.0241, 300.0270, 255.0291, 243.0292	quercetin *O*-pentosyl-hexoside		4.05 ± 0.44
51	15.23	280	759.2327	C_33_H_44_O_20_	−0.8	519.1495, 549.1604, 639.1915, 579.1704, 209.0441	(epi)catechin 3-*O*-deoxyhexosyl-hexosyl-hexoside	0.278	1.17 ± 0.02
		*–*	393.1761/439.1816 [+FA]	C_17_H_30_O_10_	1.3	149.0445, 191.0544, 251.0748, 179.0567	oxygenated megastigmane *O*-hexoside		NQ
52	15.45	270, 325	551.1774	C_26_H_32_O_13_	−0.6	389.1235, 235.0613, 431.1343, 193.0497, 165.0550, 295.0840	acylated phenylpropanoid/(neo)lignan *O*-glycoside	0.144	0.81 ± 0.01
53	15.61	254, 349	755.2043	C_33_H_40_O_20_	−0.4	299.0193, 271.0240, 609.1456, 446.0838	quercetin *O*-deoxyhexosyl-hexoside *O*-deoxyhexoside	0.096	0.61 ± 0.02
54	15.69	255, 269, 347	711.1778	C_31_H_36_O_19_	0.1	299.0197, 271.0235, 446.0857, 565.1230	quercetin glycoside	0.109	0.32 ± 0.00
55	15.92	255, 269, 345	579.1357	C_26_H_28_O_15_	−0.3	299.0199, 271.0241, 446.0857	quercetin *O*-pentoside *O*-deoxyhexoside	0.710	1.90 ± 0.02
		266, 329	901.2637	C_39_H_50_O_24_	−2.0	593.1501, 255.0302, 285.0387, 283.0242, 227.0351	kaempferol *O*-deoxyhexosyl-hexoside *O*-deoxyhexosyl-hexoside		
56	16.27	268, 326	931.252	C_43_H_48_O_23_	−0.7	283.0242, 785.1931, 255.0294, 579.1357, 430.0900	kaempferol *O*-deoxyhexosyl-hexoside *O*-feruloyl-hexoside	3.430	0.92 ± 0.01
		266, 344	593.1511	C_27_H_30_O_15_	0.1	283.0244, 593.1503, 255.0294, 227.0346	kaempferol *O*-hexoside *O*-deoxyhexoside		12.88 ± 0.92
57	16.46	255, 265, 349	609.1455	C_27_H_30_O_16_	1.0	271.0241, 300.0273, 255.0296, 243.0293	quercetin *O*-neohesperidoside#	32.159	
		265, 346	739.2078	C_33_H_40_O_19_	1.8	283.0242, 593.1503, 255.0294, 227.0346	kaempferol *O*-deoxyhexosyl-hexoside *O*-deoxyhexoside		138.41 ± 1.22
58	16.57	255, 267, 347	463.0879	C_21_H_20_O_12_	0.6	300.0273, 271.0242, 255.0293	quercetin 3-*O*-glucoside#	2.280	1.18 ± 0.12
		265, 342	695.1833	C_31_H_36_O_18_	−0.5	283.0243, 549.1249, 255.0297, 227.0347, 430.0901	kaempferol *O*-pentosyl-pentoside *O*-deoxyhexoside		4.38 ± 0.42
59	17.15	266, 332	901.2389	C_42_H_46_O_22_	2.5	283.0244, 255.0296, 755.1831, 227.0343, 579.1360, 430.0918	kaempferol *O*-deoxyhexosyl-hexoside *O*-coumaroyl-hexoside	1.629	1.56 ± 0.23
		265, 345	563.1398	C_26_H_28_O_14_	1.8	283.0244, 255.0295, 430.0901	kaempferol *O*-pentoside *O*-deoxyhexoside		14.02 ± 0.43
60	17.29	265, 345	739.2088	C_33_H_40_O_19_	0.3	283.0245, 593.1511, 255.0296, 227.0345	kaempferol *O*-deoxyhexosyl-hexoside *O*-deoxyhexoside	1.317	
		265, 345	579.1354	C_26_H_28_O_15_	0.2	255.0295, 284.0326, 227.0345, 429.0823	kaempferol *O*-pentosyl-hexoside		0.99 ± 0.05
61	17.41	254, 347	593.1509	C_27_H_30_O_15_	0.4	299.0196, 271.0246, 301.0348, 447.0926	quercetin *O*-deoxyhexoside *O*-deoxyhexoside	2.502	14.84 ± 0.28
		265, 323	871.2303	C_41_H_44_O_21_	−0.1	283.0247, 725.1723, 255.0299, 227.0350, 579.1352, 430.0908	kaempferol *O*-coumaroyl-hexosyl-pentoside *O*-deoxyhexoside		
62	17.68	254, 269, 341	769.2191	C_34_H_42_O_20_	0.7	313.0356, 623.1622, 299.0198, 271.0243	isorhamnetin *O*-neohesperidoside *O*-deoxyhexoside	1.487	
		265, 346	563.1404	C_26_H_28_O_14_	0.4	283.0248, 255.0301, 430.0900	kaempferol *O*-pentoside *O*-deoxyhexoside		0.86 ± 0.04
63	18.02	265, 341	769.2189	C_34_H_42_O_20_	1.0	313.0358, 623.1620, 299.0197, 271.0241	isorhamnetin *O*-deoxyhexosyl-hexoside *O*-deoxyhexoside	0.856	
		265, 341	593.1511	C_27_H_30_O_15_	0.1	255.0297, 284.0325, 227.0349	kaempferol *O*-deoxyhexosyl-hexoside		
		268	565.1929 [+FA]/519.1882	C_26_H_32_O_11_	0.8	329.1392, 359.1508, 297.0772, 175.0770	(neo)lignan glycoside (symplocosin-like)		NQ
64	18.29	264, 339	739.2089	C_33_H_40_O_19_	0.2	285.0400, 431.0985, 593.1518, 255.0294, 227.0344	kaempferol *O*-deoxyhexosyl-hexoside *O*-deoxyhexoside	0.337	0.98 ± 0.06
		265, 343	593.1511	C_27_H_30_O_15_	0.2	313.0353, 460.0996, 270.0171, 285.0411, 300.0266, 242.0216	kaempferol *O*-hexoside *O*-deoxyhexoside		0.76 ± 0.01
65	18.53	265, 342	447.0929	C_21_H_20_O_11_	0.8	255.0297, 227.0348, 284.0323	kaempferol *O*-hexoside (trifolin-like)	0.745	0.21 ± 0.01
		285, 300sh	597.1835	C_27_H_34_O_15_	−1.6	357.0977, 387.1088, 477.1401, 417.1189	phloretin di-*C*-hexoside		NQ
66	18.92	264, 340	739.209	C_33_H_40_O_19_	0.2	285.0404, 431.0983, 593.1529, 255.0299, 227.0350	kaempferol *O*-deoxyhexosyl-hexoside *O*-deoxyhexoside	0.842	4.19 ± 0.08
		–	547.2401	C_25_H_40_O_13_	−0.8	191.0565, 149.0451, 311.0983, 251.0768, 191.0561, 161.0452	megastigmane glycoside		NQ
67	19.05	264, 343	593.1515	C_27_H_30_O_15_	−0.5	255.0298, 285.0398, 227.0348	kaempferol *O*-hexoside *O*-deoxyhexoside	0.886	1.06 ± 0.08
68	19.21	263, 342	577.1558	C_27_H_30_O_14_	0.8	283.0246, 255.0296, 431.0978, 227.0349	kaempferol 3,7-di-*O*-deoxyhexoside (lespedin-like)	12.573	61.80 ± 0.21
69	19.35	263, 342	709.1983	C_32_H_38_O_18_	0.3	285.0401, 431.0985, 563.1408, 255.0299, 227.0347	kaempferol *O*-hexoside	2.667	3.46 ± 0.26
		263, 340	447.0927	C_21_H_20_O_11_	1.3	255.0296, 227.0349, 284.0324	kaempferol *O*-deoxyhexosyl-pentoside *O*-deoxyhexoside		
70	19.49	280, 315	653.2442	C_31_H_42_O_15_	1.3	191.0566, 149.0449, 251.0758, 311.0990, 283.0237	acylated megastigmane glycoside	0.230	NQ
71	19.62	255, 263, 343	447.0931	C_21_H_20_O_11_	0.4	271.0244, 300.0265, 255.0287, 243.0291	quercetin *O*-deoxyhexoside	0.745	1.17 ± 0.01
		*–*	547.2389	C_25_H_40_O_13_	1.3	149.0448, 191.0561, 311.0991, 251.0768, 131.0341, 221.0666	megastigmane glycoside		NQ
		255, 263, 343	477.1033	C_22_H_22_O_12_	1.2	271.0244, 243.0295, 285.0402, 314.0430, 257.0450, 299.0203	isorhamnetin *O*-hexoside		2.13 ± 0.05
72	19.81	*–*	547.2385	C_25_H_40_O_13_	2.0	251.0768, 191.0561, 221.0664, 311.0981, 149.0446, 293.0873	megastigmane glycoside	2.837	NQ
		263, 342	709.1984	C_32_H_38_O_18_	0.3	285.0401, 431.0984, 563.1406, 255.0293, 227.0344	kaempferol *O*-deoxyhexosyl-pentoside *O*-deoxyhexoside		1.12 ± 0.19
73	20.78	275	565.1929 [+FA]/519.1864	C_26_H_32_O_11_	1.5	306.0772, 339.1228, 324.1005	(neo)lignan glycoside (symplocosin-like)	0.930	NQ
74	21.82	307	429.1764	C_20_H_30_O_10_	0.5	249.1125, 205.1224, 285.0393, 227.0342, 255.0292	phenylpropanoid/(neo)lignan glycoside	0.263	0.75 ± 0.01
Total phenolics by UHPLC-UV (mg/g d.m.)									383.92
Total flavan−3-ols by UHPLC-UV (mg catechin eq./g d.m.)									30.14 ± 0.74
Total hydroxycinnamic acid derivatives by UHPLC-UV (mg chlorogenic acid eq./g d.m.)									20.18 ± 0.25
Total flavonols by UHPLC-UV (mg rutin eq./g d.m.)									333.48 ± 1.80
Total phenolic content (mg GAE/g d.m.)									255.20 ± 6.24
Condensed tannin content (mg CE/g d.m.)									74.78 ± 0.29

^a^—In order from highest to lowest intensity; ^b^—% area of all integrated peaks; ^c^—content (mg/g of dry mass, mean ± SD); ^d^—no absorption, low intensity, or coelution with other compounds; ^e^—formic acid adduct; ^f^—NQ, not quantified; ^g^—#, identity confirmed with an authentic standard.

**Table 2:** UHPLC-HR-QTOF-MS/MS characteristics and content of metabolites in *Elaeagnus multiflora* fruit extract.

No	RT (min)	UV λmax (nm)	Observed [M − H]^−^, *m*/*z*	Formula	Error (ppm)	MS/MS Fragments ^a^	Identity	CAD Area (%) ^b^	Content (mg/g d.m.) ^c^
1	0.76	– ^d^	611.1455	C_23_H_32_O_19_	1.7	306.0761, 272.0889, 338.0480, 143.0457	oxygenated oligosaccharide/organic acid glycoside derivative	27.07	NQ ^e^
		–	645.1890	C_24_H_38_O_20_	−0.3	585.1669, 513.1467, 245.0666, 377.1096	tetrasaccharide		NQ
		–	1041.3141	C_39_H_62_O_32_	1.0	981.2917, 909.2750, 245.0665, 645.1926, 305.0870	sugar oligomer		NQ
2	1.18	–	611.1455	C_23_H_32_O_19_	1.7	306.0767, 272.0887, 338.0488, 254.0785, 143.0453	oxygenated oligosaccharide/organic acid glycoside derivative	1.35	NQ
3	1.38	270	331.0671	C_13_H_16_O_10_	0.1	169.0141, 271.0456, 211.0249	galloyl *O*-hexoside	0.49	1.92 ± 0.06
4	1.46	275	282.0841	C_10_H_12_N_5_O_5_	0.9	150.0418, 195.0333, 133.0157	guanosine	1.13	NQ
		–	645.1883	C_24_H_38_O_20_	0.2	585.1685, 305.0901	tetrasaccharide		NQ
5	1.63	270	169.0142	C_7_H_6_O_5_	0.5	125.024	gallic acid# ^f^	0.99	1.84 ± 0.06
6	1.94	–	645.1883	C_24_H_38_O_20_	−0.1	585.1680, 305.0901	tetrasaccharide	1.41	NQ
		276	913.1799	C_45_H_38_O_21_	−0.1	305.0664, 423.0720, 609.1232, 727.1282, 243.0308, 177.0196	prodelphinidin trimer ((epi)gallocatechin trimer)		9.11 ± 0.05
7	2.35	277	609.1246	C_30_H_26_O_14_	0.6	423.0715, 305.0665, 177.0192, 255.0294, 441.0829	B-type prodelphinidin dimer ((epi)gallocatechin dimer)	0.62	2.56 ± 0.04
8	2.66	255	315.0719	C_13_H_16_O_9_	0.7	255.0500, 153.1899, 195.0294	dihydroxybenzoic acid *O*-hexoside	0.32	NQ
9	2.88	275	164.0715	C_9_H_10_NO_2_	1.0	147.044	phenylalanine	3.76	NQ
10	2.99	–	372.0935	C_15_H_18_NO_10_	0.2	249.0609, 122.0241	benzoxazinone hexoside	0.17	NQ
		271	783.0690	C_34_H_24_O_22_	−0.5	300.9985, 275.0192, 229.0136, 481.0619	hydrolyzable tannin (terflavin B-like)		0.77 ± 0.04
11	3.38	271	783.0684	C_34_H_24_O_22_	0.4	300.9981, 275.0187, 229.0134, 481.0626	hydrolyzable tannin (terflavin B-like)	1.25	
		275	305.0668	C_15_H_14_O_7_	2.2	125.0247, 219.0653, 261.0759, 167.0347, 179.0348, 165.0184	(epi)gallocatechin		3.39 ± 0.13
		270	331.0663	C_13_H_16_O_10_	0.8	169.0143, 125.0237	galloyl *O*-hexoside		1.30 ± 0.05
12	3.94	271	345.0820	C_14_H_18_O_10_	2.0	183.0295, 225.0398, 285.0612	methylgallate *O*-hexoside	1.03	
13	4.06	280, 317	331.0671	C_14_H_20_O_9_	0.5	153.0169, 168.0433	methoxyphenolic *O*-hexoside (leonuriside-like)	0.96	0.09 ± 0.00
14	4.24	275, 300sh	609.1246	C_30_H_26_O_14_	0.6	423.0715, 305.0665, 177.0192, 255.0294, 441.0829	B-type prodelphinidin dimer ((epi)gallocatechin dimer)	0.72	2.66 ± 0.15
		–	206.0457	C_10_H_8_NO_4_	0.9	144.0441, 134.0604, 146.0239, 162.0564	dioxindoleacetic acid		NQ
		280, 310	355.0664	C_15_H_16_O_10_	1.9	209.0293, 191.0191, 163.0397	hydroxycinnamate derivative		0.26 ± 0.00
15	4.43	271	345.0820	C_14_H_18_O_10_	1.9	183.0298, 168.0051, 124.0173	methylgallate *O*-hexoside	0.22	0.86 ± 0.06
16	4.63	271	345.0821	C_14_H_18_O_10_	1.8	183.0298, 168.0063, 124.0169	methylgallate *O*-hexoside	0.74	0.70 ± 0.03
		277	593.1305	C_30_H_26_O_13_	−0.8	305.0665, 423.0716, 255.0289, 467.0974	procyanidin/prodelphinidin dimer ((epi)catechin + (epi)gallocatechin)		3.45 ± 0.26
17	4.90	269	183.0298	C_8_H_8_O_5_	0.6	168.0059, 124.0166	methylgallic acid	0.32	1.24 ± 0.02
18	5.18	278	203.0823	C_11_H_12_N_2_O_2_	1.4	116.0504, 142.0653, 159.0927	tryptophan	3.41	NQ
		255	447.1144	C_18_H_24_O_13_	0.1	168.0059, 149.9963, 124.0163	dihydroxybenzoic acid *O*-pentosyl-hexoside		NQ
		271	633.0729	C_27_H_22_O_18_	0.7	300.9983, 463.0501, 275.0192	ellagitannin (corilagin-like)		3.08 ± 0.04
19	5.43	280, 310	355.0665	C_15_H_16_O_10_	1.6	191.0205, 209.0310, 147.0284, 163.0393	hydroxycinnamate derivative	0.22	0.29 ± 0.00
		269	183.0300	C_8_H_8_O_5_	−0.8	168.0061, 124.0166	methylgallic acid		0.46 ± 0.02
		275	380.1566	C_15_H_26_NO_10_	−1.1	267.0716, 203.0842, 146.0810, 308.1368	*N-*containing aromatic glycoside		NQ
20	5.54	273	359.0987	C_15_H_20_O_10_	−0.8	197.0452, 182.0223, 138.0315, 123.0086, 153.0556	syringic acid *O*-hexoside	0.65	NQ
		260, 290	285.0613	C_12_H_14_O_8_	1.2	152.0118, 108.0197	protocatechuic acid *O*-pentoside		NQ
21	5.81	255	447.1142	C_18_H_24_O_13_	0.4	152.0108, 271.0822, 108.0208, 315.0827	dihydroxybenzoic acid *O*-pentosyl-hexoside	0.46	NQ
		280, 310	355.0677	C_15_H_16_O_10_	−0.1	209.0297, 191.0194, 147.0325	hydroxycinnamate derivative		0.63 ± 0.01
22	5.94	274	335.1241	C_16_H_20_N_2_O_6_	2.1	203.082	tryptophan pentoside	0.74	NQ
		276, 300sh	783.0682	C_34_H_24_O_22_	0.5	300.9985, 275.0189, 229.0140, 257.0080, 481.0623	hydrolyzable tannin (terflavin B-like)		0.84 ± 0.01
		280, 310	355.0667	C_15_H_16_O_10_	1.0	209.0298, 191.0189, 147.0291	hydroxycinnamate derivative		0.16 ± 0.01
23	6.17	280, 305sh	233.1293	C_13_H_18_N_2_O_2_	1.1	119.0500, 191.1202	*p*-coumaroylputrescine	0.27	
24	6.29	325, 290	385.0777	C_16_H_18_O_11_	−0.2	191.0198, 341.0913, 209.0299, 147.0282	feruloyl hexuronic acid	0.53	0.27 ± 0.01
		300	313.0568	C_13_H_14_O_9_	−0.9	191.0215, 147.0290	salicylic acid *O*-hexuronoside		NQ
25	6.77	275	305.0660	C_15_H_14_O_7_	2.2	125.0247, 219.0653, 261.0759, 167.0347, 179.0348, 165.0184	(epi)gallocatechin	0.73	1.27 ± 0.02
26	7.01	280, 310sh	175.0609	C_7_H_12_O_5_	−1.8	115.061	unidentified conjugated organic acid	0.45	NQ
		273	359.0980	C_15_H_20_O_10_	1.0	197.0456, 239.0559, 299.0758	syryngic acid *O*-hexoside		NQ
27	7.16	309	337.1139	C_16_H_18_O_8_	0.8	191.0556, 163.0411, 119.0497, 319.1299	coumaroylquinic acid	0.35	0.10 ± 0.00
28	7.35	278	289.0714	C_15_H_14_O_6_	1.3	245.0817, 203.0706, 221.0807	catechin#	0.37	9.58 ± 0.18
		–	655.1150	C_27_H_28_O_19_	0.2	449.0726, 287.0184, 259.0230	gossypetin *O*-hexoside *O*-hexuronoside		0.23 ± 0.01
29	7.57	273, 320sh	405.0481	C_19_H_10_N_4_O_7_	−1.0	241.0018, 119.0501, 163.0395	*N-*rich aromatic metabolite	0.41	NQ
30	7.82	273	483.0777	C_20_H_20_O_14_	0.7	271.0444, 211.0244, 313.0554, 169.0134	digalloyl *O*-hexoside	0.21	0.32 ± 0.01
31	7.97	280, 310sh	598.1226	C_29_H_20_N_5_O_10_	−1.7	436.0697, 349.0377, 290.0241, 178.9983	*N-*rich aromatic metabolite	0.32	NQ
		323, 295sh	353.0877	C_16_H_18_O_9_	0.4	191.056	5-chlorogenic acid#		0.75 ± 0.01
32	8.19	–	417.1035	C_17_H_22_O_12_	0.9	152.0106, 241.0711	iridoid-/monoterpene *O*-hexoside	1.89	NQ
		271	483.0776	C_20_H_20_O_14_	0.8	211.0243, 271.0453, 169.0139, 331.0663, 313.0564	digalloyl *O*-hexoside		5.05 ± 0.02
33	8.46	310, 283	325.0924	C_15_H_18_O_8_	1.6	145.0283, 117.0341, 163.0404	coumaroyl *O*-hexoside	2.49	0.35 ± 0.02
		–	457.1710	C_21_H_30_O_11_	1.2	277.1072, 189.1281	megastigmane glycoside (amygdalactone *O*-hexoside)		NQ
34	8.65	321, 295sh	305.1614	C_15_H_22_N_4_O_3_	−2.1	289.1298, 272.1024, 247.1079, 204.0653, 190.0507	feruloylagmatine	2.78	1.26 ± 0.04
		–	443.1918	C_21_H_32_O_10_	1.2	189.1270, 237.1481	megastigmane glycoside		NQ
		272	633.0720	C_27_H_22_O_18_	2.1	300.9984, 465.0663, 275.0190	ellagitannin (corilagin-like)		1.19 ± 0.10
35	8.77	310, 283	325.0932	C_15_H_18_O_8_	−1.0	163.0395, 119.0498, 279.2324	coumaroyl *O*-hexoside	1.26	0.30 ± 0.02
		270	635.0885	C_27_H_24_O_18_	0.8	465.0671, 483.0790, 169.0139, 313.0558	trigalloyl *O*-hexoside		0.52 ± 0.07
36	8.88	271	935.0784	C_41_H_28_O_26_	−1.2	299.0191, 275.0192, 249.0402, 571.0729, 633.0723	hydrolyzable tannin (casuarinin-like)	0.75	1.02 ± 0.05
		323, 295sh	367.1032	C_17_H_20_O_9_	0.6	193.0498, 134.0364	feruloylquinic acid		0.27 ± 0.02
37	9.40	271	935.0792	C_41_H_28_O_26_	0.4	275.0188, 300.9983, 633.0724, 229.0133	hydrolyzable tannin (casuarinin-like)	1.14	1.47 ± 0.03
38	9.59	–	431.1195	C_18_H_24_O_12_	0.2	152.011	iridoid *O*-hexoside (asperulosidic acid-like)	1.15	NQ
39	9.83	270	635.0888	C_27_H_24_O_18_	0.8	465.0679, 483.0790, 169.0137, 313.0558	trigalloyl *O*-hexoside	0.78	0.74 ± 0.03
40	10.15	320	397.1139	C_18_H_22_O_10_	0.2	223.0604, 149.0232, 164.0476	sinapoylquinic acid	0.30	0.69 ± 0.02
41	10.38	–	401.1452/447.1498 [+FA] ^g^	C_18_H_26_O_10_	2.7	269.103	oxygenated megastigmane *O*-pentoside	1.01	NQ
		323, 295sh	355.1029	C_16_H_20_O_9_	1.6	175.0400, 160.0160, 193.0515, 295.0805, 217.0504	feruloyl *O*-hexoside		0.24 ± 0.01
		325, 282	371.0980	C_16_H_20_O_10_	0.9	249.0611, 121.0296	feruloyl-hexonic acid		0.08 ± 0.00
42	10.69	–	349.1868/395.1922 [+FA]	C_16_H_30_O_8_	0.3	162.0423, 179.0553	megastigmane-like/norisoprenoid *O*-hexoside	0.41	NQ
		–	583.2603 [+FA]/537.2541	C_24_H_42_O_13_	0.8	293.0859, 191.0546	megastigmane glycoside		NQ
43	10.93	257, 290	329.0881	C_14_H_18_O_9_	−0.8	167.0345, 123.0445, 191.0353, 209.0454	vanilloyl *O*-hexoside	0.69	NQ
44	11.12	283, 320sh	595.1662	C_27_H_32_O_15_	1.1	355.0815, 385.0918, 475.1241, 235.0231, 313.0700	flavanone/dihydrochalcone di-*C*-hexoside (naringenin-like)	0.24	NQ
45	11.27	–	401.1443/447.1494 [+FA]	C_18_H_26_O_10_	2.9	269.1018, 259.0597, 287.0552	oxygenated megastigmane *O*-pentoside	2.26	NQ
		323, 295sh	385.1131	C_17_H_22_O_10_	2.4	205.0498, 190.0267, 223.0599, 175.0031, 313.0568, 271.0598	sinapoyl *O*-hexoside		2.28 ± 0.01
46	11.41	323, 295sh	305.1605	C_15_H_22_N_4_O_3_	0.2	289.1303, 247.1078, 272.1028, 204.0658	feruloylagmatine	0.62	
47	11.52	273	351.1286	C_14_H_24_O_10_	3.1	249.06	unidentified oxygenated glycoside (small norisoprenoid-derived glycoside)	0.28	NQ
48	11.71	–	401.1442/447.1498 [+FA]	C_18_H_26_O_10_	2.7	269.1031, 161.0458	oxygenated megastigmane *O*-pentoside	0.63	NQ
		265, 343	755.2037	C_33_H_40_O_20_	0.4	593.1509, 283.0241, 255.0290, 227.0338	kaempferol *O*-deoxyhexoside *O*-hexoside *O*-hexoside		0.25 ± 0.00
49	12	270	635.0884	C_27_H_24_O_18_	1.0	465.0666, 169.0137, 313.0555	trigalloyl *O*-hexoside	0.76	1.84 ± 0.05
50	12.27	270	635.0883	C_27_H_24_O_18_	1.0	465.0674, 169.0138, 313.0563	trigalloyl *O*-hexoside	0.95	1.87 ± 0.07
51	12.51	–	517.2281/ 563.2336 [+FA]	C_24_H_38_O_12_	1.7	205.1226, 293.0868, 385.1857, 191.0552, 149.0439, 233.0660	megastigmane *O*-glycoside	0.88	NQ
52	12.84	325, 282	371.0978	C_16_H_20_O_10_	1.5	249.0608, 121.0289, 353.0889, 231.0498	feruloyl-hexonic acid	1.86	0.05 ± 0.00
		270	635.0893	C_27_H_24_O_18_	−0.4	465.0667, 483.0781, 169.0138, 313.0565, 125.0247, 211.0241	trigalloyl *O*-hexoside		0.89 ± 0.05
53	13.01	–	517.2278/ 563.2333 [+FA]	C_24_H_38_O_12_	2.5	205.1216, 293.0859, 233.0641, 385.1854	megastigmane *O*-glycoside	1.38	NQ
54	13.35	254, 265, 351	741.1878	C_32_H_38_O_20_	0.7	299.0192, 271.0241, 446.0844, 595.1305	quercetin *O*-pentosyl-hexoside *O*-deoxyhexoside	1.52	1.63 ± 0.01
		255, 265, 349	887.2455	C_38_H_48_O_24_	0.9	299.0188, 271.0239, 741.1872, 446.0897, 255.0284	quercetin *O*-pentosyl-deoxyhexosyl-hexoside *O*-deoxyhexoside		
		271	935.0792	C_41_H_28_O_26_	0.4	275.0188, 300.9983, 633.0724, 229.0133	hydrolyzable tannin (casuarinin-like)		0.33 ± 0.00
55	13.98	–	521.2594/567.2648 [+FA]	C_24_H_42_O_12_	1.9	389.2172, 293.0887, 227.1644, 155.1067	megastigmane *O*-pentosyl-hexoside	0.78	NQ
		321	385.1132	C_17_H_22_O_10_	1.8	267.0711, 249.0607	sinapoyl *O*-hexoside		0.13 ± 0.01
56	14.11	–	423.1863	C_18_H_32_O_11_	2.6	243.1232, 199.1332	oxygenated megastigmane *O*-hexoside	0.46	NQ
		–	563.2341 [+FA]/517.2267	C_25_H_40_O_14_	0.7	311.0983, 251.0765, 221.0663, 191.0556, 149.0443	acylated/oxygenated megastigmane glycoside		NQ
		254, 349	625.1401	C_27_H_30_O_17_	1.6	271.0236, 316.0214, 287.0188, 259.0237	myricetin 3-*O*-deoxyhexosyl-hexoside		0.52 ± 0.00
57	14.28	255, 347	621.1819	C_29_H_34_O_15_	1.0	275.0552, 305.0657, 247.0604, 219.0647, 501.1393, 381.0963	methoxyflavone di-*C*-hexoside	0.48	0.44 ± 0.00
58	14.53	265, 346	725.1922	C_32_H_38_O_19_	1.7	283.0240, 255.0294, 447.0928, 227.0346, 430.0901	kaempferol *O*-pentosyl-hexoside *O*-deoxyhexoside	0.29	0.35 ± 0.00
		265, 345	871.2497	C_38_H_48_O_23_	1.9	283.0241, 725.1926, 255.0293, 227.0343, 430.0893	kaempferol *O*-pentosyl-deoxyhexosyl-hexoside *O*-deoxyhexoside		
59	14.59	254, 265, 351	741.1874	C_32_H_38_O_20_	1.3	271.0238, 300.0271, 255.0289, 243.0296	quercetin *O*-pentosyl-deoxyhexosyl-hexoside	0.52	0.49 ± 0.00
		273, 320sh	565.2140	C_24_H_38_O_15_	−0.4	403.1599, 223.0967, 385.1473, 179.1075	acylated oxygenated norisoprenoid		NQ
60	15.01	254, 349	755.2032	C_33_H_40_O_20_	1.1	299.0187, 271.0239, 609.1457, 446.0839	quercetin *O*-deoxyhexosyl-hexoside *O*-deoxyhexoside	0.66	0.52 ± 0.00
		255, 266, 350	595.1296	C_26_H_28_O_16_	1.5	271.0241, 300.0270, 255.0291, 243.0292	quercetin *O*-pentosyl-hexoside (peltatoside-like)		0.32 ± 0.00
		273	245.0924	C_13_H_14_N_2_O_3_	3.0	203.0817, 116.0518	*N-*acetyltryptophan		NQ
61	15.15	280	759.2329	C_33_H_44_O_20_	−0.4	519.1498, 549.1600, 639.1920, 579.1709, 209.0445, 477.1392	(epi)catechin 3-*O*-deoxyhexosyl-hexosyl-hexoside	2.04	4.82 ± 0.04
		–	385.1134	C_17_H_22_O_10_	1.7	263.0762, 121.0300	iridoid hexoside (asperuloside-like)		NQ
62	15.35	273	403.1603	C_18_H_28_O_10_	1.6	241.1074, 179.1171, 223.0964	oxygenated norisoprenoid/megastigmane-like glycoside	0.24	NQ
63	15.63	257, 363	300.9991	C_14_H_6_O_8_	−0.3	283.9959, 245.0091, 257.0081, 201.0197	ellagic acid#	0.59	NQ
		254, 349	755.2034	C_33_H_40_O_20_	1.9	299.0190, 271.0247, 609.1463	quercetin *O*-deoxyhexosyl-hexoside *O*-deoxyhexoside		0.11 ± 0.00
64	15.90	310, 290	436.2233	C_25_H_31_N_3_O_4_	2.1	290.1871, 316.1656, 119.0495	*N*,*N-*di-coumaroylspermidine	0.38	NQ
65	16.21	–	365.1446	C_15_H_26_O_10_	2.0	249.0590, 115.0756, 2667.0720, 347.1344	oxygenated megastigmane glycoside	0.47	NQ
66	16.48	255, 265, 349	609.1455	C_27_H_30_O_16_	1.0	271.0241, 300.0273, 255.0296, 243.0293	quercetin 3-*O*-neohesperidoside#	5.76	0.13 ± 0.00
		265, 346	739.2076	C_33_H_40_O_19_	2.1	283.0247, 593.1508, 255.0298	kaempferol *O*-deoxyhexosyl-hexoside *O*-deoxyhexoside		0.62 ± 0.00
		271	787.0996	C_34_H_28_O_22_	0.5	617.0774, 465.0668, 169.0139, 313.0559, 635.0880, 283.0241	tetragalloyl *O*-hexoside		6.20 ± 0.02
		275	523.2174	C_26_H_36_O_11_	2.1	361.1649, 346.1410, 165.0557	phenylpropanoid/(neo)lignan glycoside		NQ
67	17.18	–	611.2558	C_26_H_44_O_16_	−0.2	431.1921, 251.1285, 207.1382	oxygenated aliphatic diglycoside/megastigmane-like diglycoside	0.25	NQ
68	17.44	273	603.2296	C_27_H_40_O_15_	−0.2	191.0559, 429.1757, 249.1124	acylated norisoprenoid/megastigmane-like glycoside	0.66	NQ
		310, 290	436.2238	C_25_H_31_N_3_O_4_	0.8	316.1665, 290.1863, 119.0498	*N*,*N-*di-coumaroylspermidine		NQ
		254, 347	593.1509	C_27_H_30_O_15_	0.4	299.0196, 271.0246, 301.0348, 447.0926	quercetin di-*O*-deoxyhexoside		0.81 ± 0.01
69	17.65	254, 269, 341	769.2185	C_34_H_42_O_20_	1.5	313.0355, 623.1608, 299.0189, 271.0245	isorhamnetin *O*-deoxyhexosyl-hexoside *O*-deoxyhexoside	0.79	
		273	603.2289	C_27_H_40_O_15_	1.0	429.1758, 249.1133, 191.0561	acylated norisoprenoid/megastigmane-like glycoside		NQ
70	17.93	273, 315	403.1605	C_18_H_28_O_10_	1.2	223.0971, 241.1235, 179.1092	oxygenated aliphatic glycoside	0.36	NQ
		280	521.2018	C_26_H_34_O_11_	1.9	329.1392, 175.0765, 359.1444, 192.0747	lignan *O*-hexoside (lariciresinol-like)		NQ
		254, 269, 341	769.2187	C_34_H_42_O_20_	1.7	313.0353, 623.1620, 299.0195, 271.0240	isorhamnetin *O*-deoxyhexosyl-hexoside *O*-deoxyhexoside		0.09 ± 0.00
71	18.39	273, 315	591.2295	C_26_H_40_O_15_	0.1	547.2763, 429.1759, 249.1125, 411.1666, 187.1130, 205.1232	acylated/oxygenated megastigmane glycoside	0.28	NQ
72	18.53	273	365.1448	C_15_H_26_O_10_	1.5	249.0610, 115.0767, 347.1337	oxygenated megastigmane glycoside	3.48	NQ
		282	597.1814	C_27_H_34_O_15_	1.8	357.0979, 387.1090, 477.1407, 417.1191	phloretin di-*C*-hexoside		NQ
73	18.99	–	547.2397	C_25_H_40_O_13_	−0.2	191.0559, 149.0449, 311.0979, 251.0758	megastigmane glycoside	0.59	NQ
74	19.17	263, 342	577.1565	C_27_H_30_O_14_	−0.4	283.0246, 255.0300, 431.0983, 227.0349	kaempferol di-*O*-deoxyhexoside (lespedin-like)	0.35	0.09 ± 0.00
75	19.54	270	939.1097	C_41_H_32_O_26_	1.3	787.0996, 617.0788, 313.0556, 169.0139, 465.0664	pentagalloyl *O*-hexoside	0.76	1.22 ± 0.05
		–	547.2388	C_25_H_40_O_13_	1.5	191.0566, 149.0453, 311.0980, 251.0768, 367.1989	megastigmane glycoside		NQ
76	19.8	–	547.2384	C_25_H_40_O_13_	1.6	251.0765, 191.0561, 221.0659, 311.0979, 149.0442, 293.0869	megastigmane glycoside	0.75	NQ
		270	939.1096	C_41_H_32_O_26_	1.4	787.0988, 617.0770, 313.0557, 169.0135, 465.0658	pentagalloyl *O*-hexoside		0.55 ± 0.03
77	20.12	270	939.1101	C_41_H_32_O_26_	0.8	787.0989, 617.0772, 313.0559, 169.0139, 465.0661	pentagalloyl *O*-hexoside	1.02	2.50 ± 0.02
78	20.28	273	603.2300	C_27_H_40_O_15_	−0.9	191.0553, 249.1124, 423.1621	acylated norisoprenoid/megastigmane-like glycoside	0.16	NQ
79	20.47	–	547.2384	C_25_H_40_O_13_	2.2	251.0768, 191.0561, 221.0664, 311.0981, 149.0446, 293.0873	megastigmane glycoside	0.18	NQ
Total phenolics by UHPLC-UV (mg/g d.m.)									90.34
Total flavan-3-ols by UHPLC-UV (mg catechin eq./g d.m.)									36.84 ± 0.38
Total hydroxycinnamic acid derivatives by UHPLC-UV (mg chlorogenic acid eq./g d.m.)									8.18 ± 0.06
Total flavonols by UHPLC-UV (mg rutin eq./g d.m.)									6.62 ± 0.02
Total gallic acid derivatives by UHPLC-UV (mg gallic acid eq./g d.m.)									38.70 ± 0.23
Total phenolic content (mg GAE/g d.m.)									490.22 ± 3.22
Condensed tannin content (mg CE/g d.m.)								855.91 ± 20.49	

^a^—In order from highest to lowest intensity; ^b^—% area of all integrated peaks; ^c^—content (mg/g of dry mass, mean ± SD); ^d^—no absorption, low intensity, or coelution with other compounds; ^e^—NQ, not quantified; ^f^—#, identity confirmed with an authentic standard; ^g^—formic acid adduct.

**Table 3:** Antioxidant activity of *Elaeagnus multiflora* Thunb. fruit and leaf extracts and reference standards dissolved in DMSO, expressed as IC_50_ values (mean ± SD).

Extract	IC_50_ µg/mL		
	DPPH	CUPRAC	ORAC
*Elaeagnus multiflora* fruit	12.7 ±0.7	11.4 ± 2.0	6.0 ± 0.7
*Elaeagnus multiflora* leaf	63.5 ± 5.4	33.1 ± 1.2	2.2 ± 0.3
Kaempferol	12.8 ± 0.5	7.1 ± 0.4	0.7 ± 0.1
Trolox^®^	8.8 ± 0.7	24.4 ± 0.2	3.0 ± 0.6

**Table 4:** IC_50_ values (µg/mL; mean ± SD) describing the antioxidant activity of *Elaeagnus multiflora* fruit and leaf extracts in liposomes prepared from erythrocyte membrane lipids (RBCLs) and erythrocyte membranes exposed to UVC radiation or AAPH. Trolox was used as a reference standard.

Extract	IC_50_ µg/mL			
	RBCLs		RBC Membrane	
	UVC	AAPH	UVC	AAPH
*Elaeagnus multiflora* fruit	21.2 ± 5.1	3.8 ± 1.2	14.8 ± 4.7	18.9 ± 2.2
*Elaeagnus multiflora* leaf	23.0 ± 6.4	9.5 ± 1.1	14.9 ± 1.0	19.7 ± 2.6
Trolox			14.6 ± 1.3 *	3.9 ± 0.3 *

* Trolox IC_50_ values were adopted from Bonarska-Kujawa et al. [9]. Trolox values were obtained using porcine erythrocyte ghost membranes under analogous experimental conditions and are provided as reference antioxidant control data. Direct quantitative comparison with extract-treated human erythrocyte ghosts and RBCL liposomes should therefore be interpreted with caution.

## Data Availability

The original data presented in the study are openly available at https://bazawiedzy.upwr.edu.pl/info/researchdata/UPWRdb7316eeaaea420f937a15ad9e386f04/ (accessed on 13 July 2026) and the corresponding authors can be contacted for requests.

## Supplementary Materials

The following supporting information can be downloaded at: https://www.mdpi.com/article/10.3390/antiox15101279/s1, Figure S1: AAPH-induced oxidation kinetics in erythrocyte-derived membrane models treated with *Elaeagnus multiflora* leaf and fruit extracts; Figure S2: UVC-induced oxidation kinetics in erythrocyte-derived membrane models treated with *Elaeagnus multiflora* leaf and fruit extracts; Figure S3: Integrated relative response patterns of *E. multiflora* leaf and fruit extracts across chemical antioxidant and membrane-related endpoints; Figure S4: Directional effect sizes for fruit versus leaf extracts across antioxidant and membrane-response endpoints; Figure S5: Statistical outcome matrix of fruit–leaf comparisons across antioxidant and membrane-response endpoints; Figure S6: Integrated Laurdan generalized polarization responses expressed as area under the concentration–response curve (AUC) in erythrocyte ghost membranes and RBCLs liposomes; Table S1: Calibration parameters and analytical validation characteristics, including linearity, calibration equation, weighting, goodness-of-fit (gof), limit of detection (LOD), limit of quantification (LOQ), and intra- and interday precision (RSD), for UHPLC–UV quantification of phenolic compounds in *Elaeagnus multiflora* Thunb. leaf and fruit extracts.
