# Supplementary material for: Phytochemical Profiles and Antioxidant Activity of Elaeagnus multiflora Thunb. Leaf and Fruit Extracts in Chemical and Erythrocyte-Derived Membrane Models
Source: Antioxidants (Basel). 2026 Oct 2;15(10):1279. doi: 10.3390/antiox15101279 (PMC13700797; doi:10.3390/antiox15101279)
Supplement: Supplementary file 1 [file antioxidants-15-01279-s001.zip › antioxidants-4554191-supplementary.pdf]

Supplementary materials

The phytochemical profile and antioxidant activity of extracts from *Elaeagnus multiflora* Thunb., and their protective effect on erythrocyte membranes.

Katarzyna Męczarska[1*], Wiktoria Tarasiuk[1], Dariusz Jedrejek [2] , Anna Bieniek[3], Sabina Lachowicz-Wisniewska[4,5,*], Dorota Bonarska-Kujawa[1] and Sylwia Cyboran-Mikołajczyk [1]

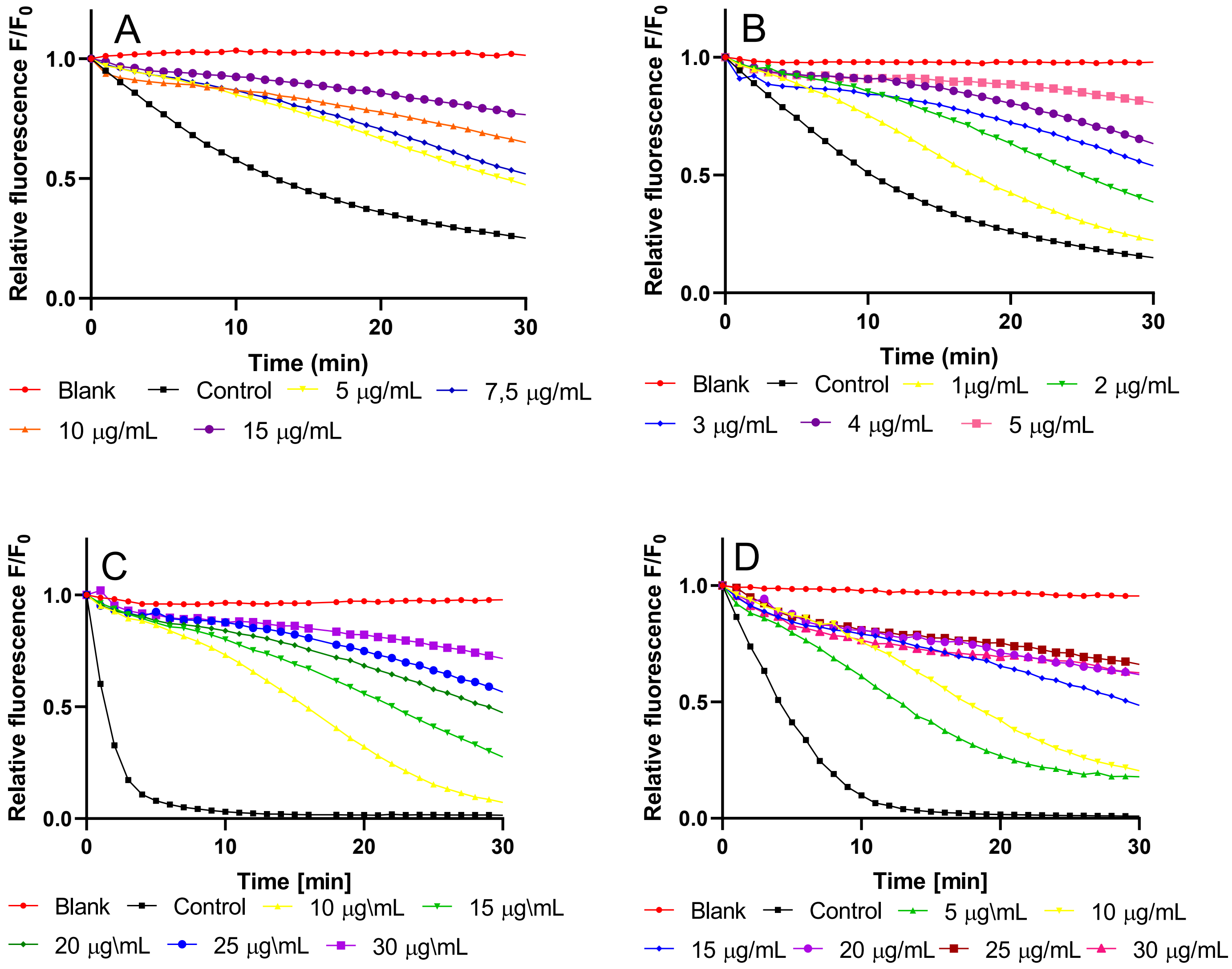

Figure S1. AAPH-induced oxidation kinetics in erythrocyte-derived membrane models treated with *Elaeagnus multiflora* leaf and fruit extracts. Representative time-dependent changes in TMA-DPH relative fluorescence intensity during AAPH-induced oxidation in (A) RBCL liposomes treated with leaf extract, (B) RBCL liposomes treated with fruit extract, (C) erythrocyte ghost membranes treated with leaf extract, and (D) erythrocyte ghost membranes treated with fruit extract.

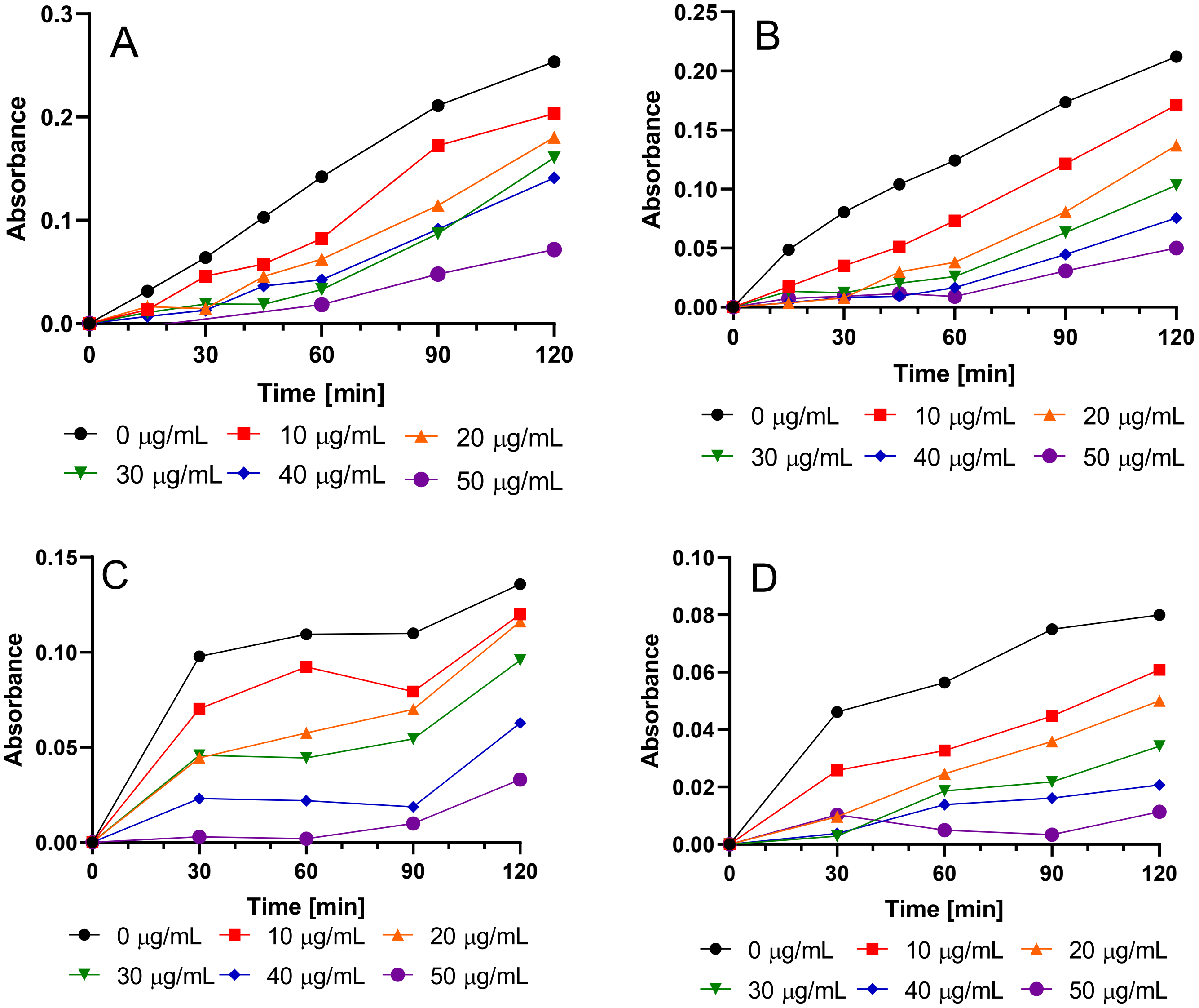

Figure S2. UVC-induced oxidation kinetics in erythrocyte-derived membrane models treated with *Elaeagnus multiflora* leaf and fruit extracts. Representative time-dependent changes in the oxidation-related absorbance signal during UVC exposure in (A) RBCL liposomes treated with leaf extract, (B) RBCL liposomes treated with fruit extract, (C) erythrocyte ghost membranes treated with leaf extract, and (D) erythrocyte ghost membranes treated with fruit extract.

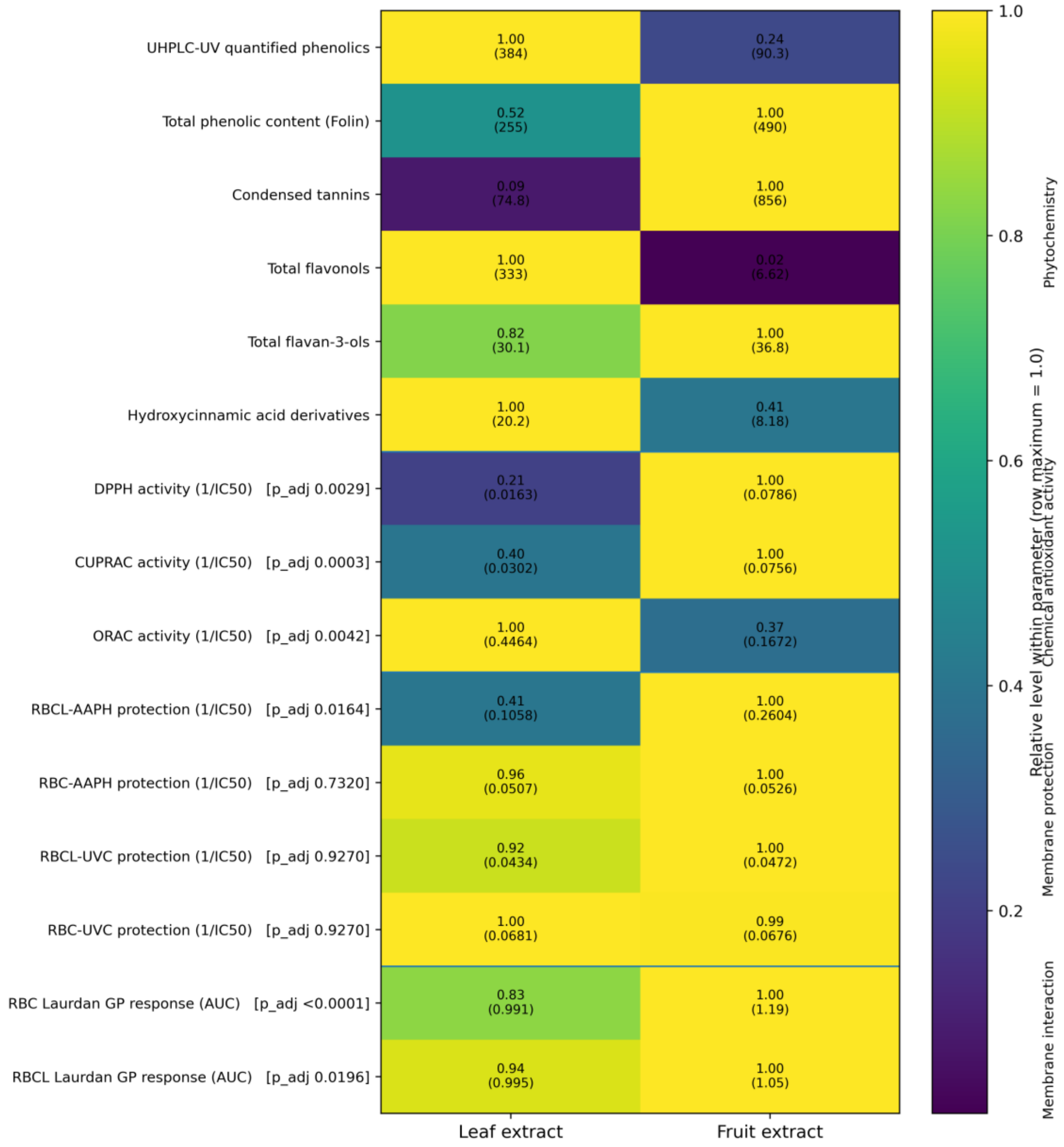

Figure S3: Integrated relative response patterns of *Elaeagnus multiflora* leaf and fruit extracts across antioxidant and membrane-related endpoints. Heatmap showing within-parameter normalized responses of the leaf and fruit extracts. Each endpoint was oriented so that higher values correspond to greater activity or response and subsequently normalized to the higher value within each parameter (maximum = 1.0). The heatmap is descriptive and was not used for inferential statistical testing.

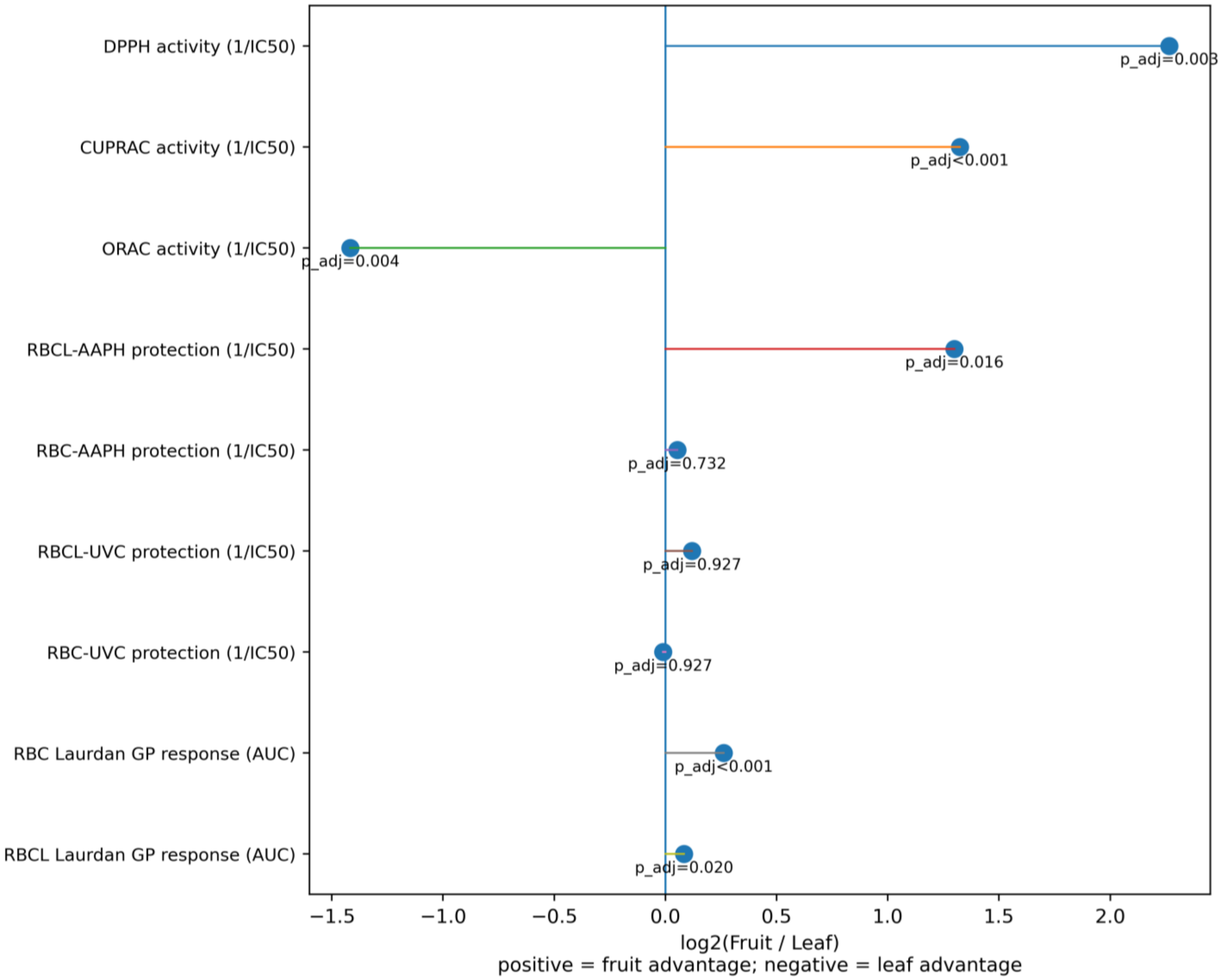

Figure S4: Directional effect sizes for fruit versus leaf extracts across antioxidant and membrane-response endpoints. Directional differences are expressed relative to the orientation of the endpoints, so that higher values consistently represent greater antioxidant activity, membrane protection, or membrane response. Positive values indicate a fruit-extract advantage, whereas negative values indicate a leaf-extract advantage.

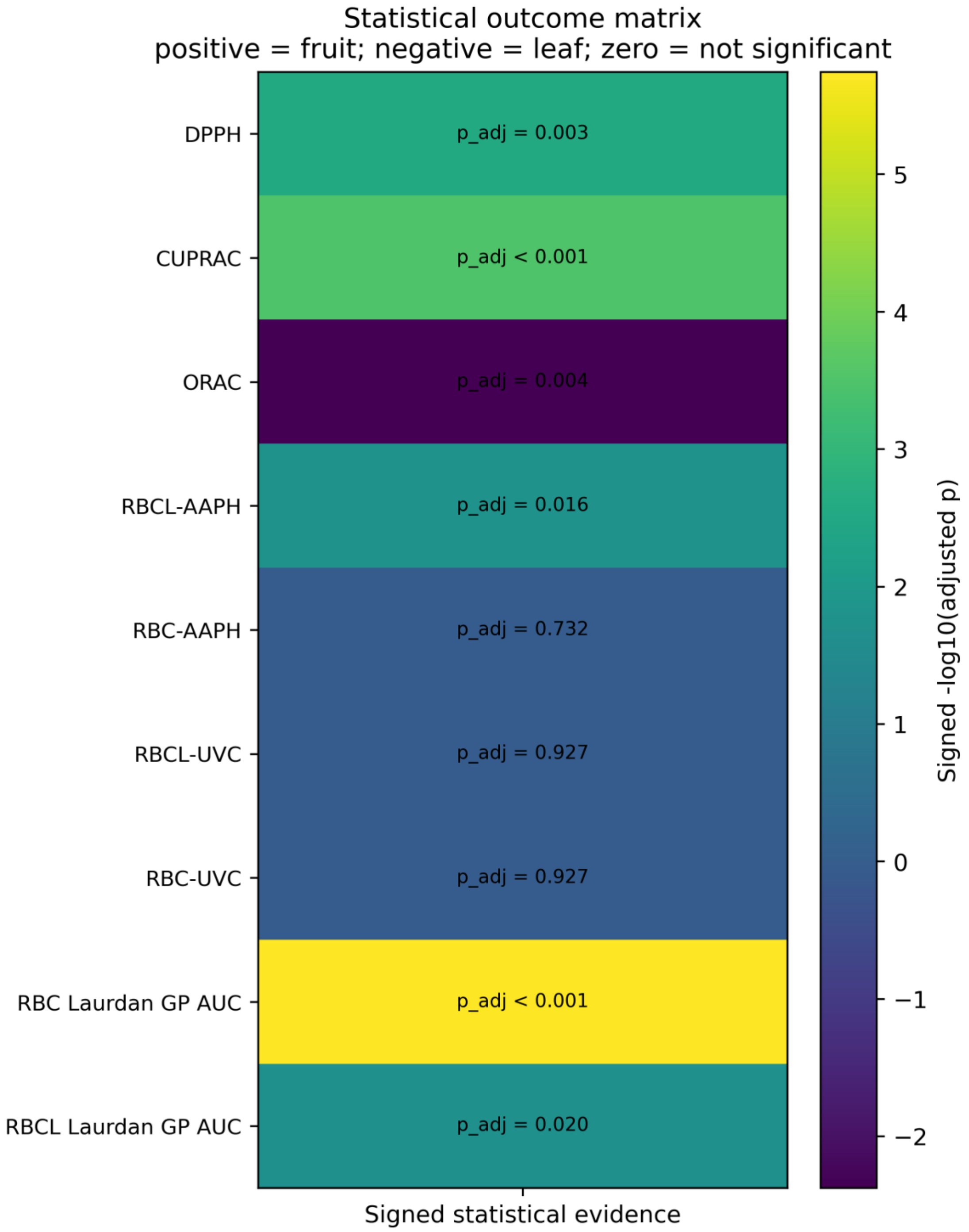

Figure S5: Statistical outcome matrix of fruit–leaf comparisons across antioxidant and membrane-response endpoints. Statistical evidence is expressed as signed $-\log_{10}(p_{adj})$, with the sign determined by the direction of the fruit-to-leaf effect. Comparisons that remained non-significant after Holm correction are shown as zero.

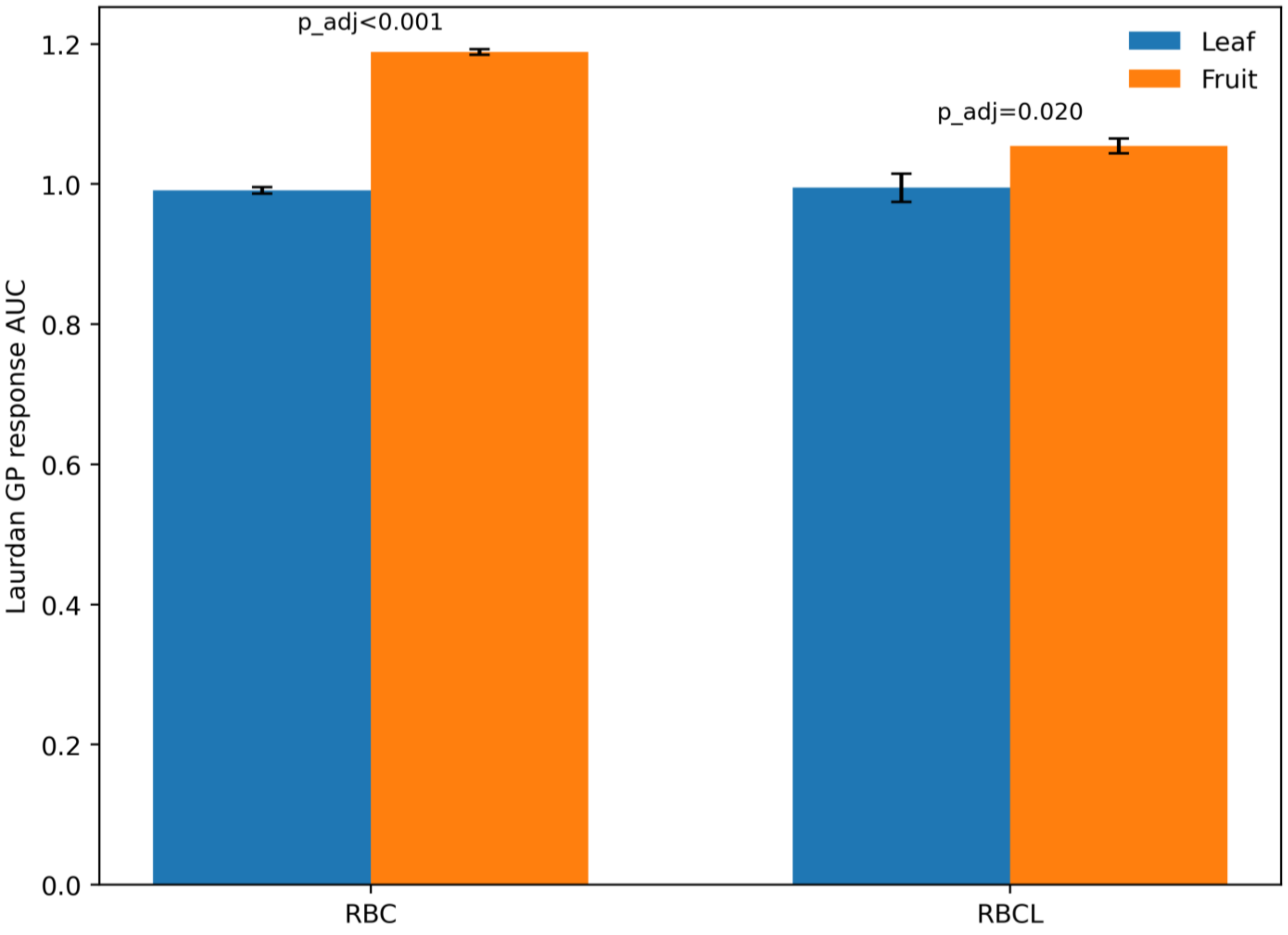

Figure S6: Integrated Laurdan generalized polarization responses expressed as area under the concentration–response curve (AUC) in erythrocyte ghost membranes and RBCL liposomes;

Table S1. Calibration parameters and analytical validation characteristics, including linearity, calibration equation, weighting, goodness-of-fit (gof), limit of detection (LOD), limit of quantification (LOQ), and intra- and interday precision (RSD), for UHPLC–UV quantification of phenolic compounds in *Elaeagnus multiflora* Thunb. leaf and fruit extracts.

Analyte	Calibration range (µg/mL)	Equation	Weighting	R^2	gof (%) (*n*=5)[a]	LOD (µg/mL)[b]	LOQ (µg/mL)[b]	RSD (%) intraday (*n*=5) / interday (*n*=10)[c]
catechin	1.0–100.0	y = 95.2797x + 0.0863	none	0.9999	0.99 ± 0.82	0.127	0.385	0.59 / 1.58
rutin	0.5–200.0	y = 243.0691x + -6.5653	1/x	0.9999	0.40 ± 0.23	0.143	0.433	0.15 / 1.08
gallic acid	0.5–75.0	y = 472.8960x + 4.0859	1/x	0.9998	0.67 ± 0.47	0.141	0.426	0.17 / 1.05
5-chlorogenic acid	0.25–50.0	y = 444.3955x + -21.1810	1/x	0.9998	0.36 ± 0.17	0.068	0.206	0.06 / 0.97

[a] – gof (%) was calculated according to the formula $g = \sqrt{\Sigma(\%\ \text{deviation})^2/(n-1)}$, with %deviation = ($x_{\text{calculated conc}} - x_{\text{nominal value}}/x_{\text{nominal value}}$) × 100. The gof (%) should fall within the range ≤ 10%.

[b] – LOD (µg/mL) and LOQ (µg/mL) were estimated from the calibration data acc. to the equations $LOD = 3.3\sigma/m$ and $LOQ = 10\sigma/m$, where σ is the SD of the intercepts from calibration curves and m is the slope of the corresponding calibration curves.

[c] – RSD (%) was calculated from the analyte response (peak area), which was analyzed five times during the day (intraday) and ten times over two days (interday).
